# Japanese Society for Cancer of the Colon and Rectum (JSCCR) guidelines 2019 for the treatment of colorectal cancer

**DOI:** 10.1007/s10147-019-01485-z

**Published:** 2019-06-15

**Authors:** Yojiro Hashiguchi, Kei Muro, Yutaka Saito, Yoshinori Ito, Yoichi Ajioka, Tetsuya Hamaguchi, Kiyoshi Hasegawa, Kinichi Hotta, Hideyuki Ishida, Megumi Ishiguro, Soichiro Ishihara, Yukihide Kanemitsu, Yusuke Kinugasa, Keiko Murofushi, Takako Eguchi Nakajima, Shiro Oka, Toshiaki Tanaka, Hiroya Taniguchi, Akihito Tsuji, Keisuke Uehara, Hideki Ueno, Takeharu Yamanaka, Kentaro Yamazaki, Masahiro Yoshida, Takayuki Yoshino, Michio Itabashi, Kentaro Sakamaki, Keiji Sano, Yasuhiro Shimada, Shinji Tanaka, Hiroyuki Uetake, Shigeki Yamaguchi, Naohiko Yamaguchi, Hirotoshi Kobayashi, Keiji Matsuda, Kenjiro Kotake, Kenichi Sugihara, Japanese Society for Cancer of the Colon and Rectum

**Affiliations:** 1 Department of Surgery, Teikyo University School of Medicine, 2-11-1 Kaga, Itabashi-ku, Tokyo, 173-8606 Japan; 2 Department of Clinical Oncology, Aichi Cancer Center Hospital, Nagoya, Japan; 3 Endoscopy Division, National Cancer Center Hospital, Tokyo, Japan; 4 Department of Radiation Oncology, Showa University School of Medicine, Tokyo, Japan; 5 Division of Molecular and Diagnostic Pathology, Graduate School of Medical and Dental Sciences, Niigata University, Niigata, Japan; 6 Department of Gastroenterological Oncology, Saitama Medical University International Medical Center, Saitama, Japan; 7 Hepato-Biliary-Pancreatic Surgery Division, Artificial Organ and Transplantation Division, Department of Surgery, Graduate School of Medicine, The University of Tokyo, Tokyo, Japan; 8 Division of Endoscopy, Shizuoka Cancer Center, Shizuoka, Japan; 9 Department of Digestive Tract and General Surgery, Saitama Medical Center, Saitama Medical University, Saitama, Japan; 10 Department of Chemotherapy and Oncosurgery, Tokyo Medical and Dental University Medical Hospital, Tokyo, Japan; 11 Department of Surgical Oncology, Graduate School of Medicine, The University of Tokyo, Tokyo, Japan; 12 Department of Colorectal Surgery, National Cancer Center Hospital, Tokyo, Japan; 13 Department of Gastrointestinal Surgery, Tokyo Medical and Dental University, Tokyo, Japan; 14 Department of Radiation Oncology, faculty of Medicine, University of Tsukuba, Ibaraki, Japan; 15 Department of Clinical Oncology, St. Marianna University School of Medicine, Kawasaki, Japan; 16 Department of Gastroenterology and Metabolism, Hiroshima University Hospital, Hiroshima, Japan; 17 Department of Gastroenterology and Gastrointestinal Oncology, National Cancer Center Hospital East, Chiba, Japan; 18 Department of Clinical Oncology, Faculty of Medicine, Kagawa University, Kagawa, Japan; 19 Division of Surgical Oncology, Department of Surgery, Nagoya University Graduate School of Medicine, Aichi, Japan; 20 Department of Surgery, National Defense Medical College, Saitama, Japan; 21 Department of Biostatistics, Yokohama City University School of Medicine, Yokohama, Japan; 22 Division of Gastrointestinal Oncology, Shizuoka Cancer Center, Shizuoka, Japan; 23 Department of Hepato-Biliary-Pancreatic and Gastrointestinal Surgery, School of Medicine, International University of Health and Welfare, Narita, Japan; 24 Department of Surgery, Institute of Gastroenterology, Tokyo Women’s Medical University, Tokyo, Japan; 25 Center for Data Science, Yokohama City University, Yokohama, Japan; 26 Division of Clinical Oncology, Kochi Health Sciences Center, Kochi, Japan; 27 Department of Endoscopy, Hiroshima University Hospital, Hiroshima, Japan; 28 Department of Specialized Surgeries, Tokyo Medical and Dental University, Tokyo, Japan; 29 Department of Gastroenterological Surgery, Saitama Medical University International Medical Center, Hidaka, Japan; 30 Library of SEIREI, SAKURA Citizen Hospital, Sakura, Japan; 31 Department of Surgery, Mizonokuchi Hospital, Teikyo University School of Medicine, Kanagawa, Japan; 32 Department of Surgery, Sano City Hospital, Tochigi, Japan; 33 Tokyo Medical and Dental University, Tokyo, Japan

**Keywords:** Colorectal cancer, Guideline, Surgery, Chemotherapy, Endoscopy, Radiotherapy

## Abstract

The number of deaths from colorectal cancer in Japan continues to increase. Colorectal cancer deaths exceeded 50,000 in 2016. In the 2019 edition, revision of all aspects of treatments was performed, with corrections and additions made based on knowledge acquired since the 2016 version (drug therapy) and the 2014 version (other treatments). The Japanese Society for Cancer of the Colon and Rectum guidelines 2019 for the treatment of colorectal cancer (JSCCR guidelines 2019) have been prepared to show standard treatment strategies for colorectal cancer, to eliminate disparities among institutions in terms of treatment, to eliminate unnecessary treatment and insufficient treatment and to deepen mutual understanding between healthcare professionals and patients by making these guidelines available to the general public. These guidelines have been prepared by consensuses reached by the JSCCR Guideline Committee, based on a careful review of the evidence retrieved by literature searches and in view of the medical health insurance system and actual clinical practice settings in Japan. Therefore, these guidelines can be used as a tool for treating colorectal cancer in actual clinical practice settings. More specifically, they can be used as a guide to obtaining informed consent from patients and choosing the method of treatment for each patient. Controversial issues were selected as clinical questions, and recommendations were made. Each recommendation is accompanied by a classification of the evidence and a classification of recommendation categories based on the consensus reached by the Guideline Committee members. Here, we present the English version of the JSCCR guidelines 2019.

Introduction

1. Guideline objectives

According to the Vital Statistics of Japan, the number of deaths from colorectal cancer in Japan has continued to increase. In 2016, the number of deaths from colorectal cancer exceeded 50,000. Many new treatment methods have been developed and their use in combination with advances in diagnostic methods has led to a steady improvement in the results of treatment. However, there are differences in treatment among medical institutions in Japan that provide medical care for patients with colorectal cancer, and the differences may lead to differences in the results of treatment.

Under such circumstances, the JSCCR guidelines 2019 for the treatment of colorectal cancer (JSCCR guidelines 2019), which are intended for doctors (general practitioners and specialists) who provide medical care for patients with colorectal cancer in various disease stages and conditions, have been prepared for the following purposes: (1) to show standard treatment strategies for colorectal cancer, (2) to eliminate disparities among institutions in terms of treatment, (3) to eliminate unnecessary treatment and insufficient treatment, and (4) to deepen mutual understanding between healthcare professionals and patients by making these guidelines available to the general public [1].

The following are expected to be achieved with these guidelines: (1) improvement of the treatment of colorectal cancer in Japan; (2) improvement of the results of treatment; (3) reduction of the human and financial burden; and (4) increased benefits for patients.

2. How to use these guidelines

These guidelines were prepared by consensuses reached by the Guideline Committee of the Japanese Society for Cancer of the Colon and Rectum, based on a careful review of the evidence retrieved by the literature searches and in view of the medical health insurance system and actual clinical practice settings in Japan and, therefore, these guidelines can be used as a tool for treating colorectal cancer in actual clinical practice settings. More specifically, they can be used as a guide to obtaining informed consent from patients and choosing the method of treatment for each patient. However, these guidelines provide only general recommendations for choosing treatment strategies for colorectal cancer, and they do not control or limit treatment strategies or treatment methods that are not described herein. They can also be used as a document to explain the rationale for selecting treatment strategies and treatment methods that differ from those described therein.

The Japanese Society for Cancer of the Colon and Rectum (JSCCR) is responsible for the statements in these guidelines. However, the personnel directly in charge of treatment, not the JSCCR or the Guideline Committee, are responsible for the outcome of treatment.

3. Users

The users of these guidelines are mainly clinical doctors engaged in all aspects of the medical treatment of colorectal cancer.

4. How to develop these guidelines

(1) Recording methods

We adopted the concept from the first edition, in which the treatment policy algorithm was disclosed, a simple explanation thereof recorded, and added further comments with regard to categories requiring additional explanation. Since the 2009 edition, areas of debate have been raised as clinical questions (CQs) and included with recommendations added. In the 2016 edition, systemic therapy was the only treatment to be revised. In the 2019 edition, all aspects of the treatments were revised, with corrections and additions made to the CQs based on knowledge acquired since the 2016 version (systemic therapy) and the 2014 version (other treatments).

Efforts were made to make the expression of the CQs clear and unambiguous. When comparing multiple interventions, we did not stick to ranking everything, and kept the expression flexible to ensure that it is useful in clinical practice. The clinicopathological terms conformed to those described in the “Japanese Classification of Colorectal, Appendiceal, and Anal Carcinoma, third English edition [2].

(2) Evidence level/strength of recommendations of CQs

The recommendations added to CQs included the evidence level and strength of recommendations determined using the following direction.

(2-1) Evidence level

Papers relating to the CQs were comprehensively collected, and the evidence indicated by individual papers relating to the critical outcomes included within the CQs was divided into groups by study design [3]. The literature level and a body of evidence (Table 1) were evaluated in reference to the GRADE* System [4–26], before determining the final CQ evidence level (Table 2).

**Table 1:**
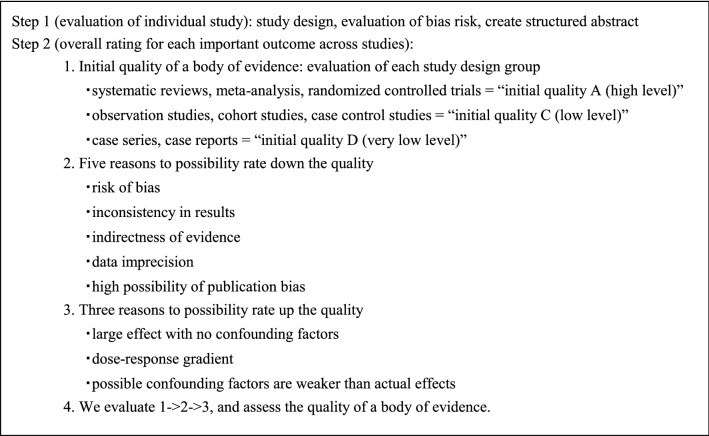
Rating the quality of evidence

**Table 2:** Definition of levels of evidence (Ref. [14])

A (high)	We are very confident in the effect estimate
B (moderate)	We are moderately confident in the effect estimate: the true effect is likely to be close to the estimate of the effect, but there is a possibility that it is substantially different
C (low)	Our confidence in the effect estimate is limited: the true effect may be substantially different from the estimate of the effect
D (very low)	We have very little confidence in the effect estimate: the true effect is likely to be substantially different from the estimate of effect

*GRADE: The Grading of Recommendations Assessment, Development and Evaluation

(2-2) Strength of recommendations

Draft recommendation statements and the strength of the recommendations were directed based on the outcomes and the level of evidence obtained from the process described above and were evaluated at a consensus meeting of the Guideline Committee. In the CQ text, the recommendations that were decided have been directly expressed, and ambiguous expressions were excluded.

The draft recommendations were evaluated from four categories (① Quality of evidence, ② Patients’ views and preferences, ③ Benefits and harms, and ④ Cost effectiveness). The strength of recommendation (Table 3) was determined by vote, based on the GRADE Grid method [11].

**Table 3:** Strength of recommendation (Ref. [25])

Strength of recommendation	
1 (Strong recommendation)	Strong “For” an intervention
	Strong “Against” an intervention
2 (Weak recommendation)	Weak “For” an intervention
	Weak “Against” an intervention

Method

1. We selected one of the following five options and voted.

① Strong “For” intervention

② A Weak “For” intervention

③ Weak “Against” intervention

④ Strong “Against” intervention

⑤ Not graded

2. With one vote, if 70% or more of the votes were obtained in any of ① to ⑤, it was considered a final decision.

If this criterion cannot be met, then the following shall be applied:

- If ① + ② exceeds 50%, ③ + ④ is 20% or lower, “weakly recommend to perform.”
- If ③ + ④ exceeds 50%, ①+ ② is 20% or lower, “weakly recommend not to perform.”

3. Items not reaching consensus after a single vote were debated once again, with the results of the first vote disclosed and additional information on the situation relating to clinical practice in Japan provided, and discussion and voting was repeated.

4. If agreement was not reached, even in the second vote, no strength of recommendation was presented in the CQ.

5. Literature search

At first, the literature search was performed for the clinical questions. Then, a further search was done as needed with additional search techniques.

To survey the latest literature, in addition to the papers used for reference in the previous edition, the PubMed and Ichushi-Web databases were selected for the search, and the English and Japanese literature was searched in both databases from June 2012 to February 2017. However, the start of the search period for systemic therapy was August 2016. The task of searching was performed by a medical librarian, who created a search formula based on a discussion with the Committee members in charge of each item and collected literature during the search period. In addition, secondary sources such as UpToDate and literature collected by manual searching were added and critically examined as needed, and other documents such as proceedings and guidelines were included as necessary. We selected 3,295 documents from among the 16,341 documents (PubMed 9,672, ICHUSHI 6,153, hand search 516) collected during the literature search and critically reviewed all of them (Table 4).

**Table 4:** Number of scientific articles retrieved and selected

	Number of articles retrieved		Number of articles selected		Number of articles retrieved manually
	PubMed	Ichushi	PubMed	Ichushi	
(1) Endoscopic treatment	1102	539	136	73	81
(2) Surgical treatment	3351	2521	926	192	82
(3) Radiotherapy	1225	181	271	16	67
(4) Systematic therapy	2019	1381	591	108	242
(5) Others	1975	1530	374	86	44
Total	9672	6153	2304	475	516

Treatment guidelines for colorectal cancer

Chapter 1: Treatment strategies for Stage 0 to Stage III colorectal cancer

1. Endoscopic treatment (Fig. 1)

**Fig. 1:**
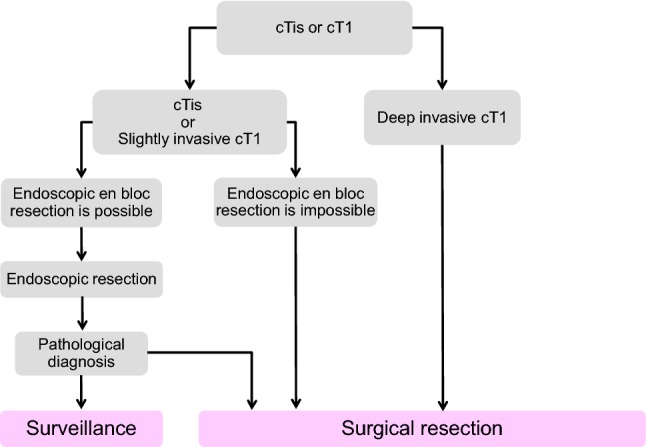
Treatment strategies for cTis and cT1 colorectal cancer

General principles underlying the indications for endoscopic resection

- There is little possibility of lymph node metastasis, and the size and location of the tumor make en bloc resection possible.
  Indication criteria for endoscopic resection:
  1. Intramucosal carcinoma or carcinoma with slight submucosal invasion
  2. Size does not matter
  3. Any macroscopic type
- Endoscopic treatment is a method of endoscopically resecting lesions in the large bowel and of collecting the resected specimens.
- Endoscopic treatment methods consist of polypectomy (note 1), endoscopic mucosal resection (EMR) (note 2), and endoscopic submucosal dissection (ESD) (note 3).
- In determining the indication for endoscopic treatment and the treatment method, information on the size, predicted depth of invasion, and morphology of the tumor is essential.

Comments

① Endoscopic resection is intended for both diagnosis and treatment. It consists of total excisional biopsy in which curability and the necessity of additional intestinal resection are assessed by histopathological examination of the resected specimens (CQ-1).
② cT1 deeply invasive cancer is diagnosed based on endoscopic findings, such as “fullness, erosion, ulcer, fold convergence, deformity, rigidity,” as well as contrast X-ray, chromoendoscopy, image-enhanced endoscopy (*e.g.*, NBI/BLI [27], or magnifying endoscopic observation) and endoscopic ultrasound findings. [28–30].
③ En bloc resection is desirable for accurate diagnosis of the status of carcinoma invasion in the resection margin and the deepest area.

- 2 cm is the largest size of a tumor that can be easily resected en bloc by polypectomy or snare EMR [31] (CQ-2).
- Colorectal ESD is an “endoscopic resection technique which enables en bloc resection of a tumor, regardless of size,” which was approved for implementation under health insurance in April 2012 with regard to “early-stage malignant tumors”. Given the high likelihood of technically difficult complications (perforations), however, it should only be implemented after sufficient consideration of the level of skill of the endoscopist performing the procedure. Tumors with a diameter between 2 and 5 cm were covered by insurance. The revision of April 2018 eliminated the upper limit of the tumor diameter and the indication became early colon cancer with a maximum diameter of 2 cm or more. Early colon cancer accompanying fibrosis is even applied to tumors with a diameter of 2 cm or less (CQ-2).
- EMRC (EMR using a cap) is reported to involve a high risk of perforation when used for colon lesions.
- If the preoperative diagnosis is cancer accompanied by adenoma (intramucosal carcinoma), a piecemeal resection can be performed with regard to the adenoma, while avoiding division of the cancerous area. It should be noted, however, that piecemeal resection is associated with a high incomplete resection rate and a high local recurrence rate. Multiple-piecemeal resection, which makes accurate histological judgment difficult, should be avoided [31].
- After endoscopic resection, the resection margin should be observed in detail and the presence or absence of a residual lesion should be confirmed.
- Dye spray and magnifying observation are useful for the diagnosis of residual lesions [30].
- If residual mucosal lesions are present, additional treatment (e.g., endoscopic additional resection, hot biopsy, cautery, etc.) should be performed.

④ Follow-up observation after endoscopic treatment

- For piecemeal resection of pTis carcinoma with a positive horizontal margin, the presence or absence of local recurrence is investigated by colonoscopy at around 6 months (CQ-3).
- For follow-up observation of pT1 cancer, a search not only for local recurrence but also lymph node recurrence and distant metastasis recurrence is necessary, with follow-ups including endoscopic examinations, image diagnoses such as CT examinations and tumor markers (CQ-3).
- While recurrence after endoscopic treatment for pT1 cancer is often within three years, caution is required as it may also recur thereafter [32].

Note 1: Polypectomy—In this technique, a snare is placed on the stalk of the lesion, and the lesion is electrocauterized using a high-frequency current. This method is mainly used for protruding lesions.
Note 2: EMR—In this technique, the lesion is elevated by local injection of a liquid such as physiological saline into the submucosa, and the lesion is electrocauterized the same as in case of polypectomy. This method includes the snare method and EMR using a cap (EMRC). It is mainly used for superficial tumors and large sessile lesions.
Note 3: ESD—In this technique, the lesion is elevated by local injection of a liquid such as sodium hyaluronate solution into the submucosa of the perilesional area; then, circumferential incision of the mucosa surrounding the lesion and dissection of the submucosa with a special knife, and en bloc resection are performed [33]. ESD is mainly indicated for large tumors, especially for early cancers, that cannot be resected by en bloc EMR.
Note 4: Precutting EMR—In this technique, snaring is performed without dissecting the submucosal layer after incising the circumference of the lesion alone, using a knife for ESD or the tip of a snare.
Note 5: Hybrid ESD—In this technique, the submucosal layer is dissected and snaring is carried out after the ESD procedure (mucosal incision + submucosal dissection, using a knife for ESD or the tip of a snare).

2. Surgical treatment (Fig. 2)

**Fig. 2:**
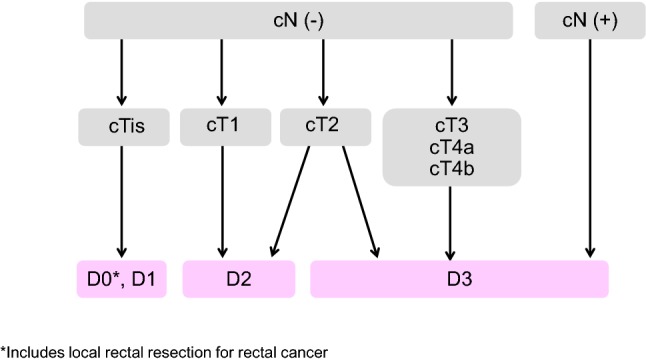
Surgical treatment strategies for cStage 0 to cStage III colorectal cancer

Principles of surgery

- The extent of lymph node dissection to be performed during colorectal cancer surgery is determined based on the preoperative clinical findings and on the extent of lymph node metastasis and depth of tumor invasion by the tumor observed intraoperatively.
- If lymph node metastasis is recognized, or suspected based on the preoperative/intraoperative findings, D3 dissection is performed [34].
- If no lymph node metastases are observed based on the preoperative/intraoperative diagnostic findings, lymph node dissection is performed based on the depth of tumor invasion [35].

1. Lymph node dissection is unnecessary for pTis cancer (D0), because pTis cancer is not accompanied by lymph node metastasis. However, D1 dissection can be performed when bowel resection is adopted.
2. D2 dissection is necessary for pT1 cancer, because the incidence of lymph node metastasis is approximately 10% and because approximately 2% of pT1 cancer is accompanied by intermediate lymph node metastasis (Table 5).

**Table 5:** Incidences of lymph node metastasis according to tumor location and depth of tumor invasion

		No. of patients	Extent of lymph node metastasis detected histologically				
			*n*_0_ (%)	*n*_1_ (%)	*n*_2_ (%)	*n*_3_ (%)	*n*_4_ (%)
All sites	sm	3151	90.7	7.3	1.9	0.0	0.1
	mp	3590	77.3	17.4	4.2	0.9	0.3
	ss/a_1_	11,272	54.6	29.9	12.0	2.3	1.2
	se/a_2_	6101	35.9	34.4	20.2	5.7	3.8
	si/ai	1502	43.0	27.6	16.4	6.7	6.3
	Total	25,617	57.1	26.3	11.9	2.9	1.9
Colon	sm	1957	91.4	6.8	1.8	0.0	0.0
	mp	1747	79.3	16.3	3.5	0.6	0.3
	ss/a_1_	7333	56.6	28.1	11.7	2.4	1.2
	se/a_2_	3363	37.4	34.0	19.3	5.6	3.7
	si/ai	960	44.6	28.6	14.7	5.5	6.6
	Total	15,360	58.6	25.4	11.3	2.8	1.8
Rectosigmoid	sm	337	88.7	9.5	1.8	0.0	0.0
	mp	429	80.4	17.0	2.6	0.0	0.0
	ss/a_1_	1584	53.9	33.0	10.2	1.3	1.7
	se/a_2_	789	34.2	38.4	20.8	3.2	3.4
	si/ai	187	44.9	24.6	19.3	4.8	6.4
	Total	3326	55.7	29.3	11.4	1.6	2.0
Upper and	sm	839	89.7	7.7	2.0	0.1	0.4
lower rectum	mp	1373	73.9	19.2	5.4	1.4	0.1
	ss/a_1_	2310	48.8	33.3	14.2	2.7	1.0
	se/a_2_	1904	33.9	33.6	21.5	6.8	4.1
	si/ai	328	38.1	26.2	19.8	10.4	5.5
	Total	6754	54.3	27.0	13.3	3.6	1.8
Anal canal	sm	18	94.4	0.0	5.6	0.0	0.0
	mp	41	70.7	9.8	7.3	7.3	4.9
	ss/a_1_	45	60.0	22.2	8.9	6.7	2.2
	se/a_2_	46	32.6	21.7	23.9	15.2	6.5
	si/ai	27	33.3	25.9	14.8	18.5	7.4
	Total	177	54.8	17.5	13.0	10.2	4.5

(JSCCR colorectal cancer registry: patients in years 2000–2004) Depth of invasion and the degree of lymph node metastasis were determined according to the rules set forth in the “Japanese Classification of Colorectal Carcinoma” (6th edition). *sm* submucosa, *mp* muscularis propria, *ss* subserosa, *se* serosa, *a1* shallow part of adventitia, *a2* deeper part of adventitia, *si/ai* direct invasion of other organs through the serosa/adventitia
3. Although there is insufficient evidence describing the extent of lymph node dissection for cT2 (MP) cancer, at least D2 dissection is necessary. However, D3 dissection can be performed, because about 1% of cT2 (MP) cancer is accompanied by main lymph node metastases (Table 5) and because preoperative diagnosis of depth of invasion is not very accurate.

For details of lateral lymph node dissection in rectal cancer, see (CQ-5).

Surgical treatment for rectal cancer:

- The principle for radical surgery for rectal cancer is TME (total mesorectal excision) or TSME (tumor-specific mesorectal excision) [36–39].

[Indication criteria for sphincter preserving surgery]

- Sphincter preserving surgery is indicated only when the following criteria are fulfilled: (i) resection with no oncologic remnant (both the distal and circumferential resection margins are negative = DM 0, RM 0) can be achieved, and (ii) the postoperative anal function can be maintained.

[Autonomic nerve-preserving surgery]

- Considering factors such as the degree of cancer progression and the presence or absence of macroscopic nerve invasion, preservation of autonomic nerves is attempted to preserve urinary and sexual functions as much as possible, provided that curability is unaffected.

[Indications criteria for lateral lymph node dissection]

- Lateral lymph node dissection is indicated when the lower border of the tumor is located distal to the peritoneal reflection and the tumor has invaded beyond the muscularis propria [40] (Table 6) (CQ-5).

**Table 6:** Lateral dissection and lateral metastasis of rectal cancer

		No. of patients	No. of patients who underwent lateral dissection	Lateral dissection rate (%)	No. of patients with lateral metastasis	Lateral metastasis rate (percentage of all patients) (%)	Lateral metastasis rate (percentage of patients who underwent lateral dissection) (%)
RS	sm	124	0	0	0	0.0	0.0
	mp	127	6	4.7	0	0.0	0.0
	ss/a_1_	316	24	7.5	0	0.0	0.0
	se/a_2_	177	8	4.5	0	0.0	0.0
	si/ai	32	14	43.8	1	3.1	7.1
	Total	776	52	6.7	1	0.1	1.9
Ra	sm	138	5	3.6	0	0.0	0.0
	mp	149	18	12.1	0	0.0	0.0
	ss/a_1_	230	58	25.2	4	1.7	6.9
	se/a_2_	181	59	32.6	7	3.9	11.9
	si/ai	15	8	53.3	0	0.0	0.0
	Total	713	148	20.8	11	1.5	7.4
RaRb + Rb	sm	234	37	15.8	2	0.9	5.4
	mp	372	218	58.6	20	5.4	9.2
	ss/a_1_	350	230	65.7	28	7.7	12.2
	se/a_2_	412	319	77.4	75	18.0	23.5
	si/ai	59	48	81.4	17	28.8	35.4
	Total	1427	852	59.7	142	9.8	16.7

(Project study by the JSCCR: patients in years 1991–1998). *RS* rectosigmoid, *Ra* upper rectum, *Rb* lower rectum

Laparoscopic surgery:

- The indications for laparoscopic surgery are determined by considering the surgeon’s experience and skills as well as tumor factors, such as the location and degree of progression of the cancer, and patient factors, such as obesity and history of open abdominal surgery (CQ-4).

Comments

[Optimal length of the bowel resection]

① In D1, D2, D3 dissection, the resection margin of the bowel is determined so that the pericolic/perirectal lymph node, as defined in *Japanese Classification of Colorectal, Appendiceal, and Anal Carcinoma* [2], is dissected.
② The extent of the pericolic/perirectal lymph node in colon cancer is defined by the positional relationship between the primary tumor and the feeding artery. Metastasis of the pericolic/perirectal lymph node at a distance of 10 cm or more from the tumor edge is rare [41]. Currently, as a JSCCR research project, a multicenter cohort study investigating the distance between metastasis-positive pericolic/perirectal lymph node and the primary tumor is ongoing.
③ The extent of the pericolic/perirectal lymph nodes in rectal cancer is defined as follows: the oral side is defined by the lowest plunge point of the sigmoid artery, while the anal side is defined by the distance from the tumor edge. For cStage 0–III cases, it is rare for intramural and/or mesorectal distal cancer spread to develop at a distance of 3 cm or more from the tumor edge in RS and Ra cancer, or 2 cm or more in Rb cancer [42–45]. Thus, the distal resection margin of the bowel and mesorectum should be determined to include this range.
④ It should be noted that pT4, pN2, M1 (Stage IV), and poorly differentiated rectal cancer cases are frequently accompanied by distal spread a long distance from the primary tumor edge [41, 43–45].

[TME/TSME]

- Total mesorectal excision (TME) is a procedure that resects all the mesorectum just above the anal canal [36]. Tumor-specific mesorectal excision (TSME) is a procedure for partially resecting the mesorectum according to the location of the tumor [39].

[Intersphincteric resection (ISR)]

- ISR is a procedure for lower rectal cancer located close to the anus, to ensure the adequate distal margin via the removal of the internal anal sphincter and to avoid a permanent stoma.
- The indication criteria for ISR are as follows: (1) able to ensure the resection with clear circumferential surgical resection margin (no infiltration to the external anal sphincter or levator ani muscles); and (2) able to ensure the adequate distal surgical margin (in general, 2 cm or more for T2/T3 tumors and 1 cm or more for T1 tumors). ISR is not recommended for cases with poorly differentiated cancer and cases in which the anal sphincter tonus is decreased.
- In a systematic review of 14 papers, the R0 resection rate of patients who underwent ISR was 97.0%, the anastomotic leakage rate was 9.1%, and the local recurrence rate was 6.7%, which is reported as an acceptable result [46]. However, according to the questionnaire survey conducted by the JSCCR in 2125 cases, the 5-year survival rate of patients who underwent ISR was equivalent to that of the lower rectal cancer cases in the JSCCR colorectal cancer registry, but the 5-year local recurrence rate (including recurrence in the area of anastomosis) was relatively high at 11.5%. Obviously, the local recurrence rate becomes higher as the depth of invasion reaches deeper (4.2% at T1, 8.5% at T2, 18.1% at T3, and 36.0% at T4). The indication of ISR should be determined based on a precise preoperative diagnosis of the tumor depth.
- As the extent of resection of the anal sphincter becomes wider, postoperative defecatory dysfunction (e.g., fecal incontinence) becomes a more serious problem. In particular, it has been reported that the incidence of defecatory dysfunction is high in patients who receive preoperative radiation therapy, those with anastomotic leakage, and the elderly [47–49].
- The indication of ISR should be carefully decided because the procedure is associated with a high degree of difficulty and has a great influence on the patient’s QOL, including the postoperative defecatory function. In addition to tumor factors (e.g., the histological type and depth), and patient factors (e.g., age and sphincter tonus), the experience and skill of the operator should be taken into consideration.

[Autonomic nerve-preserving surgery]

- The autonomic nervous system related to surgery for rectal cancer consists of the lumbar splanchnic nerves*, superior hypogastric plexus*, hypogastric nerves*, pelvic splanchnic nerves#, and pelvic plexus. (*sympathetic nerves, #parasympathetic nerves)
- Regarding the urinary function, if one side of the pelvic nerve plexus is preserved [AN 1–4], a certain function is maintained.
- The hypogastric nerve controls the ejaculation function, and the internal pelvic nerve governs the erectile function. To maintain the male sexual function, full conservation of the autonomic nervous system on both sides [AN 4] is necessary.
- The urinary function and male sexual function may be impaired even if the autonomic nervous system is fully preserved, regardless of whether lateral lymph node dissection is performed or not [50–52].

[Local excision for rectal cancer]

- Local excision is indicated for cTis cancer and cT1 cancer (slight invasion) located distal to the second Houston valve (peritoneal reflection).
- Histological investigation of the resected specimen allows a determination to be made of the likelihood that treatment will cure the condition completely, along with the need for additional treatment (intestinal resection accompanied by lymph node dissection).

[Aggregate data from the JSCCR colorectal cancer registry]

① The incidence of lymph node metastasis according to site and depth of tumor invasion, curative resection rate, and 5-year survival rate are shown in Tables 5, 7, and 8 [35].

**Table 7:** Curative resection rate according to pStage (lower rows: no. of patients)

pStage	I	II	IIIa	IIIb	IV	All stages
All patients	98.7%	96.2%	91.9%	81.8%	−	78.0%
	5455	7336	5635	2572	4300	25,298
Colon	99.1%	96.6%	92.4%	83.6%	−	77.2%
	3028	4688	3208	1379	2787	15,090
Rectosigmoid	99.5%	96.6%	92.5%	80.2%	−	78.0%
	615	961	835	288	560	3259
Upper and lower rectum	97.9%	95.0%	90.9%	80.5%	−	79.9%
	1764	1644	1564	866	929	6767
Anal canal	95.8%	86.0%	78.6%	61.5%	−	70.9%
	48	43	28	39	24	182

(JSCCR colorectal cancer registry: patients in years 2000–2004)

Curative resection rate = Number of patients with histological curability A cancer/Total number of patients who underwent surgery

Staging was performed according to the rules set forth in the “Japanese Classification of Colorectal Carcinoma” (6th edition)

**Table 8:** Cumulative 5-year survival rate according to tumor location (lower row**s**: no. of patients)

pStage	0	I	II	IIIa	IIIb	IV	All Stages
Cecum	91.0%	93.7%	83.5%	73.0%	65.4%	12.5%	68.2%
	79	185	249	207	113	204	1037
Ascending colon	93.9%	91.2%	85.8%	79.1%	63.4%	19.1%	71.4%
	125	338	656	416	211	410	2156
Transverse colon	88.9%	91.4%	85.2%	78.5%	65.7%	20.8%	74.0%
	105	277	428	244	138	210	1402
Descending colon	100.0%	94.1%	85.3%	82.0%	52.9%	21.1%	75.4%
	43	146	224	166	52	117	748
Sigmoid colon	94.2%	92.3%	85.8%	83.0%	64.7%	22.0%	73.7%
	154	852	1124	837	363	736	4066
Rectosigmoid	89.4%	91.5%	84.8%	78.0%	60.0%	19.8%	71.6%
	54	366	539	473	175	322	1929
Upper rectum	98.0%	95.3%	84.6%	75.9%	57.7%	11.6%	72.4%
	67	356	464	471	173	263	1794
Lower rectum	97.5%	88.3%	81.7%	70.0%	51.4%	11.6%	70.5%
	142	718	486	473	332	298	2449
Anal canal	100.0%	78.7%	90.9%	46.9%	61.2%	15.7%	60.0%
	4	16	14	16	19	17	86
Colon	93.0%	92.3%	85.4%	80.4%	63.8%	19.9%	72.8%
	506	1798	2681	1870	877	1677	9409
Rectum (Ra + Rb)	97.6%	90.6%	83.1%	73.0%	53.5%	14.8%	71.3%
	209	1074	950	944	505	561	4243
All sites	94.0%	91.6%	84.8%	77.7%	60.0%	18.8%	72.1%
	773	3254	4184	3303	1576	2577	15,667

(JSCCR colorectal cancer registry: patients in years 2000–2004)

Only adenocarcinomas (including mucinous carcinomas and signet-ring cell carcinomas) were counted

Survival rates were calculated by the life table method with death from any cause as an event

5-year censoring rate = 20.5% (3208/15,667)

Staging was performed according to the rules set forth in the “Japanese Classification of Colorectal Carcinoma” (6th edition)
② The 5-year survival rates after curative resection of pStage 0 to pStage III colorectal cancer according to site were: All sites: 82.2%, Colon: 83.8%, Rectosigmoid: 81.7%, Ra–Rb rectum: 79.3% (patients in years 2000–2004).

Chapter 2: Treatment strategies for Stage IV colorectal cancer (Fig. 3)

**Fig. 3:**
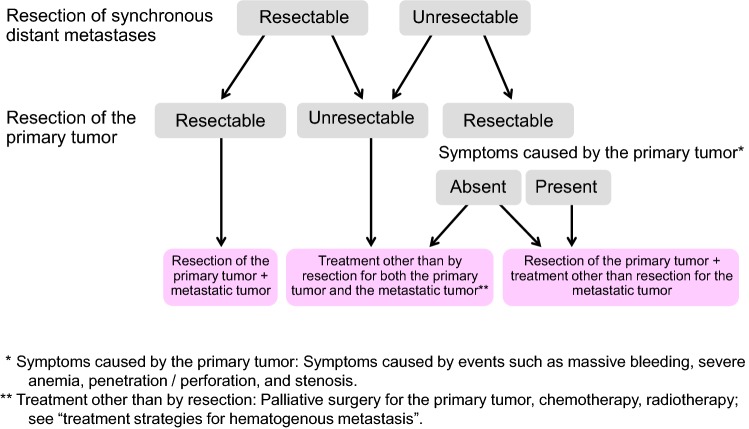
Treatment strategies for Stage IV colorectal cancer

- Stage IV colorectal cancer is associated with synchronous distant metastasis to any of the following organs: liver, lung, peritoneum, brain, distant lymph nodes, or other organ (e.g., bone, adrenal gland, spleen).
- If both the distant metastases and the primary tumor are resectable, curative resection of the primary tumor is performed, and resection of the distant metastases is considered.
- If the distant metastases are resectable but the primary tumor is unresectable, in principle, resection of the primary tumor and distant metastases is not performed, and another treatment method is selected.
- If the distant metastases are unresectable but the primary tumor is resectable, the indication for the resection of the primary tumor is determined, based on the clinical symptoms of the primary tumor and the impact on the prognosis (CQ-6).

Comments

① The incidence of synchronous distant metastasis is shown in Table 9.

**Table 9:** Incidence of synchronous distant metastasis of colorectal cancer

	Liver	Lung	Peritoneum	Other sites				
				Bone	Brain	Virchow	Other	Total
Colon cancer	11.8%	2.2%	5.7%	0.3%	0.0%	0.1%	1.3%	1.8%
No. of patients 15,391	1815	338	875	47	6	23	205	281
Rectal cancer	9.5%	2.7%	2.6%	0.5%	0.0%	0.1%	1.1%	1.7%
No. of patients 10,221	970	273	266	49	5	6	112	172
Total no. of pateints	10.9%	2.4%	4.5%	0.4%	0.0%	0.1%	1.2%	1.8%
25,621	2785	611	1141	96	11	29	317	453

(JSCCR colorectal cancer registry: patients in years 2000–2004)
② Liver metastases
  If resectable, liver metastases should be resected upon confirming the radicality of the primary resection.
  As for the timing of resection, simultaneous resection of the primary lesion and liver metastases can be safely performed [53]. Depending on the difficulty of hepatectomy and the general condition of the patient, metachronous resection is also performed. However, it is unclear whether simultaneous resection or metachronous resection improves the long-term prognosis.
③ Lung metastases
  If resectable, resection of lung metastases should be considered after resection of the primary tumor.
  Metachronous resection is generally performed to remove lung metastases after primary resection.
④ Peritoneal metastases (CQ-7)
  Complete resection is strongly recommended for P1.
  Complete resection is recommended for P2 when easily resectable.
  The efficacy of resection of P3 has not been demonstrated.
⑤ Distant lymph node metastases

Excision of distant lymph node metastases may be considered, but no comparative clinical trials have shown a clear therapeutic effect. However, in recent years, resection of para-aortic lymph node metastases was reported to have the potential to achieve radical cure and longer survival at certain rates.

Excision of distant lymph node metastases may be considered, but no comparative clinical trials have shown a clear therapeutic effect. However, in recent years, resection of para-aortic lymph node metastases was reported to have the potential to achieve a radical cure and longer survival at certain rates [54–58].

⑥ Other distant metastases (bone, brain, adrenal gland, spleen, etc.)
  Although there are reports of resection of these metastatic lesions, no clear effect on survival has been shown.
⑦ Cases accompanied by distant metastasis to multiple organs
  Typically, these cases involve metastasis to the liver or lungs.
  If it is safe and simple to remove the primary lesion and the metastasized lesions in the liver or lungs, resection should also be considered [59, 60] (CQ-8).
⑧ Adjuvant therapy subsequent to the resection of distant metastasis
  Although evidence is lacking with regard to the efficacy of adjuvant chemotherapy, in view of the high recurrence rate, it is recommended that adjuvant chemotherapy should be performed after the curative resection of distant metastasis (CQ-19).

Chapter 3: Treatment strategies for recurrent colorectal cancer (Fig. 4)

**Fig. 4:**
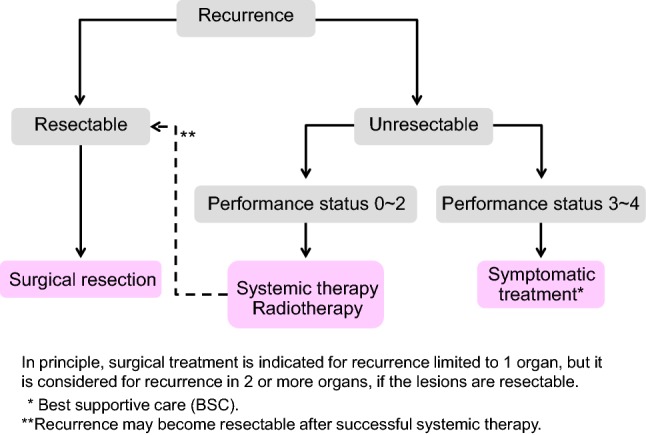
Treatment strategies for recurrent colorectal cancer

- The goal of treatment for recurrent colorectal cancer is improvement of the prognosis and patient’s QOL.
- Treatment methods include surgery, systemic therapy, and radiotherapy. Arterial infusion chemotherapy and thermal ablation therapy are not recommended (CQ-13, 24).
- An appropriate treatment method should be selected with the informed consent of the patient in view of a variety of factors, such as the prognosis, complications, and QOL expected after treatment.
- If recurrence is observed in a single organ and complete surgical resection of the recurrent tumor (s) is possible, resection is strongly considered.
- If recurrence is observed in more than a single organ, resection can be considered if the recurrent tumors in all the organs are resectable [59, 61]. The efficacy of curative resection in patients who have liver and lung metastases has been shown and, thus, resection should be considered (CQ-8).
- Some authors believe that resection of liver or lung metastases should be performed only after a certain observation period to rule out occult metastases [62, 63].
- Systemic therapy is effective with regard to cases of unresectable liver metastasis, with some cases demonstrating that curative resection may become possible [64, 65] (CQ-10).
- The efficacy and safety of preoperative chemotherapy for resectable recurrent lesions are not clear, and application should be considered with caution (CQ-9).
- For adjuvant chemotherapy following resection of recurrent colorectal cancer, there is no clear evidence of efficacy with the exception of reports showing that 5-FU or UFT/LV prolongs the relapse-free survival after resection of liver metastasis (CQ-19).

Comments

[Treatment methods for hematogenous metastases] (See Chapter 4 “Treatment strategies for hematogenous metastases”)

[Lymph node recurrence/peritoneal recurrence]

① In general, it is reasonable to regard lymph node recurrence or peritoneal recurrence after curative resection of the primary tumor as a part of systemic disease. Thus, systemic therapy should be conducted referring to the section on systemic therapy for unresectable colorectal cancer (See Chapter 5.2. Systemic therapy for unresectable colorectal cancer).
② Resection for localized lymph node recurrence or peritoneal recurrence could be considered only when the disease is controlled. However, its efficacy is not clear. The surgical indication should be decided after careful consideration of the risk of surgery and the postoperative quality of life [54, 57, 66–68].
③ Radiotherapy may be effective for treating localized lymph node recurrence [69–71].

[Local recurrence of rectal cancer]

① The extent of spread of the recurrent tumor is evaluated by diagnostic imaging, and resection is considered only for patients in whom complete resection can be expected, after taking into consideration such factors as the pattern of recurrence, symptoms, and physical findings (CQ-14).
② The indication for the palliative resection of local recurrence for the purpose of improving survival and providing relief from symptoms should be carefully considered because its effectiveness is not established [72].
③ If complete resection cannot be expected, systemic therapy is the first choice of treatment from the viewpoint of continuous disease control. However, local effects, such as the alleviation of symptoms, can be expected from radiation therapy. Chemoradiotherapy or radiotherapy can also be a treatment option if symptoms, effects, and adverse events are fully considered [73] (CQ-26).

Chapter 4: Treatment strategies for hematogenous metastases (Fig. 5)

**Fig. 5:**
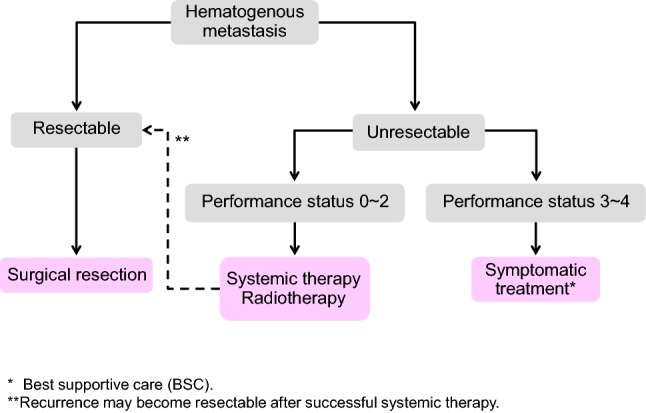
Treatment strategies for hematogenous metastases

1. Treatment strategies for liver metastases

- Treatment of liver metastases is broadly divided into hepatectomy, systemic therapy, hepatic arterial infusion therapy, and thermal ablation therapy.
- Hepatectomy is recommended for liver metastases when curative resection is possible.
- Hepatectomy consists of systematic resection and partial (non-systematic) resection.
- Indication criteria for hepatectomy

1. The patient is capable of tolerating surgery
2. The primary tumor has been controlled or can be controlled.
3. The metastatic liver tumor can be completely resected.
4. There are no extrahepatic metastases or they can be controlled.
5. The function of the remaining liver will be adequate.
  Systemic therapy is considered for patients with unresectable liver metastases whose general condition can be maintained at a certain level or higher (PS 0 to PS 2).
  Thermal ablation therapy consists of microwave ablation therapy (MCT) and radiofrequency ablation (RFA).
  If the patient’s general condition is poor (PS ≥ 3), or there is no effective chemotherapy, best supportive care (BSC) is provided.

Comments

[Hepatectomy]

① The efficacy of hepatectomy is not based on evidence derived from a cohort study or a randomized controlled trial. However, good results have been shown that were not obtained with other treatments.
② The 5-year survival rate after hepatectomy is 35–58% [74–77]. In a multicenter study conducted in Japan, the 3-year survival rate of the 585 patients who received hepatectomy was 52.8% and the 5-year survival rate was 39.2% [78].
③ Whether or not the complete resection of the metastatic lesion is possible is determined based on the comprehensive assessment of the number, size, location of metastatic lesions and the predicted residual liver volume.
④ The sensitivity of magnetic resonance imaging (MRI) in detecting lesions of < 10 mm in size has been reported to be significantly higher than CT [79]. The efficacy of positron emission tomography (FDG-PET) in the diagnosis and treatment of liver metastasis is not established [80].
⑤ Excision without exposure of the cancer to the resected stump is important [81–84].
  There are reports that recommend a resection margin distance of 1 cm or more [85, 86] and that state that no cancer exposure is sufficient [87–90].
⑥ In synchronous liver metastasis, excision of the primary tumor may be performed prior to the excision of liver metastasis, which may be excised after the evaluation of the radicality of the primary tumor. There is no clear conclusion as to the timing of resection of synchronous liver metastasis [91–93].
⑦ Since the prognosis in the case of hepatic hilar lymph node metastasis is poor, there is a report that hepatic hilar lymph node metastasis is regarded as a contraindication of hepatectomy [94–96].
  In Japan, the 5-year survival rate after the dissection of hepatic hilar lymph node metastasis is reported to be 12.5% [78].
⑧ There are reports showing the efficacy of hepatectomy in patients who have controllable extrahepatic metastases (mainly lung metastases) in addition to liver metastases [59–61, 97] (CQ-8).
⑨ A 5-year survival rate of 21–52% has been reported in cases involving rehepatectomy for residual liver recurrence. For residual liver recurrence cases, resection should be considered in light of the above-mentioned indications for hepatectomy [84, 98–106].
⑩ Evidence to support the efficacy of adjuvant chemotherapy after hepatectomy is not sufficient; however, implementation is recommended in view of the high rate of recurrence (CQ-19).
⑪ The safety of preoperative chemotherapy for resectable liver metastases has not been established (CQ-9).

[Treatment methods other than resection]

① Systemic therapy is performed for patients with unresectable liver metastases.
② In cases of inoperable liver metastasis, hepatic arterial infusion therapy or thermal ablation therapy is not generally recommended (CQ-13, CQ-24).
③ In Japan, there are no data to support the efficacy of stereotactic body radiation therapy or brachytherapy.
④ If the patient’s general condition is poor, an appropriate BSC is provided.

2. Treatment strategies for lung metastases

- Treatment of lung metastases consists of pneumonectomy and systemic therapy, and radiotherapy.
- Pneumonectomy is considered if the metastatic lung tumor is resectable.
- Pneumonectomy consists of systematic resection and partial (non-systematic) resection.

Indication criteria for pneumonectomy

1. The patient is capable of tolerating surgery.
2. The primary tumor has been controlled or can be controlled.
3. The metastatic lung tumor can be completely resected.
4. There are no extrapulmonary metastases or they can be controlled.
5. The function of the remaining lung will be adequate.

- Systemic chemotherapy is considered for patients with unresectable lung metastases whose general condition can be maintained at a certain level or higher.
- Even if the patient cannot tolerate surgery, stereotactic body radiation therapy is considered if the primary tumor and extrapulmonary metastases are controlled or can be controlled and the number of lung metastases within 5 cm in diameter is no more than three [107].
- If the patient’s general condition is poor, an appropriate BSC is provided.

Comments

[Pneumonectomy]

① The efficacy of lung resection is not based on evidence derived from a cohort study or randomized controlled trial. However, good results that have not been obtained with other treatments have been shown in appropriately selected patients [97, 108–115].
② The 5-year survival rate after pulmonary resection is 30–68% [116–118]. In the multicenter aggregate conducted in the JSCCR project study, the 5-year survival rate of lung resection cases was 46.7% and the cumulative 5-year relapse-free survival rate was 33.7%, while the 5-year survival rate of non-resected cases was 3.9% [116, 119].
③ In synchronous pulmonary metastasis, it is desirable to initially resect the primary lesion and evaluate local curability. Thus, in principle, metachronous resection is performed for synchronous pulmonary metastasis.
④ The number, size, location, and intra-bronchial development of metastatic lesions should be evaluated, and a procedure that enables the complete resection of the metastatic lesion with secure resection margins should be decided.
⑤ The significance of hilar/mediastinal lymph node dissection is not established. The number of metastases, bilateral lung metastasis, hilar/mediastinal lymph node metastasis, serum CEA value before lung resection, primary factor (T factor, N factor), and disease-free interval (DFI) is reported to be poor prognostic factors [112–116, 120].
⑥ In cases of controllable extrapulmonary metastasis (mainly liver metastasis), there are reports suggesting the efficacy of lung resection [60, 97, 113, 115, 121, 122].
⑦ A five-year survival rate of 20–48% has been reported in patients who undergo repeat lung resection for residual lung recurrence [112, 114, 115, 123, 124]. Even for residual lung recurrence after lung resection, the indications for resection should be carefully considered according to the above-mentioned indication criteria for lung resection.
⑧ No large-scale studies have examined the efficacy of adjuvant chemotherapy after the curative resection of lung metastases (CQ-19).

3. Treatment strategies for brain metastases

- Brain metastases are often detected as a part of a systemic disease, and surgical therapy or radiotherapy is considered for lesions in which treatment can be expected to be effective.
- The optimal treatment method is selected after considering the patient’s general condition and status of other metastatic tumors, and evaluating the size and location of metastatic brain tumors and the number of brain lesions.
- Radiotherapy is considered for patients with unresectable metastases.

[Surgical therapy]

Indications criteria for brain resection [125, 126]

1. The patient is capable of tolerating surgery.
2. The primary tumor has been controlled or can be controlled.
3. The patient has a life expectancy of at least several months.
4. Resection will not cause significant neurologic symptoms.
5. There are no metastases to other organs or they can be controlled.

[Radiotherapy]

- The purpose of radiotherapy is to relieve symptoms, such as cranial nerve symptoms and intracranial hypertension symptoms, and to prolong survival time by reducing locoregional relapse.
- Whole-brain radiotherapy is considered for patients with multiple brain metastases and for patients with a solitary brain metastasis for which surgical resection is not indicated.
- Stereotactic irradiation is considered when the number of brain metastases is about no more than three or four and the maximum diameter of each metastasis does not exceed 3 cm.

Comments

[Surgical therapy]

① Approximately, 90% of cases of brain metastasis involve metastasis to other organs, and the prognosis is poor, even if resection is performed [125, 127–131].
② The average survival time after excision of solitary brain metastasis is reported to be 30–40 weeks [125, 126, 128, 129, 132]. However, the efficacy of surgical therapy has not been determined based on the evaluation of a suitably sized cohort.
③ The significance of adding whole-brain radiotherapy after brain metastasis resection is controversial [125].

[Radiotherapy]

① The symptom improvement rate is 60–80% [133, 134].
② Stereotactic irradiation achieves a local control rate of 80–90% [135].
③ According to a systematic review, the median survival time after stereotactic irradiation, whole-brain radiotherapy, and BSC was 6.4 months, 4.4 months, and 1.8 months, respectively [136].
④ Age, PS, number of brain metastases, and control of extracranial lesions have been reported as prognostic factors [137–139].
⑤ At present, whole-brain radiotherapy is performed irrespective of the number of metastases. When a prognosis of several years can be expected, whole-brain radiotherapy in combination with stereotactic irradiation is considered [140, 141]. In the case of stereotactic irradiation, single treatment is also considered as a treatment option as it can achieve a high QOL. However, surveillance at appropriate intervals by image inspection is necessary because the rate of intracranial recurrence is higher in comparison to that after whole-brain radiotherapy.

4. Treatment strategies for hematogenous metastases to other organs

- Resection is also considered for other hematogenous metastases, such as to the adrenal glands, skin, and spleen, if they are resectable. However, patients with such metastases often have metastasis to more than one organ, and chemotherapy or radiotherapy is often indicated.

Chapter 5: Systemic therapy

- Systemic therapy consists of adjuvant chemotherapy to prevent postoperative recurrence and systemic therapy to treat unresectable colorectal cancer.
- Commonly used anticancer drugs that have been approved for the indication of colorectal cancer and are covered by the Japanese National Health Insurance include the followings:

Cytotoxic drugs: fluorouracil (5-FU), 5-FU + levofolinate calcium (*l*-LV), tegafur uracil (UFT), tegafur gimeracil oteracil potassium (S-1), UFT + calcium folinate (LV), capecitabine (Cape), irinotecan hydrochloride hydrate (IRI), oxaliplatin (OX), trifluridine/tipiracil hydrochloride (FTD/TPI), etc.

Molecular targeted drugs: bevacizumab (BEV), ramucirumab (RAM), aflibercept beta (AFL), cetuximab (CET), panitumumab (PANI), regorafenib hydrate (REG)

Immune checkpoint inhibitor: pembrolizumab (Pembro)

1. Adjuvant chemotherapy

- Postoperative adjuvant chemotherapy is a systemic chemotherapy that is performed after surgery to prevent recurrence and improve the prognosis of patients who have undergone R0 resection.

General principles for the indications of adjuvant chemotherapy

1. Stage III colorectal cancer (colon and rectal cancer) for which R0 resection has been performed.
2. The patient has recovered from postoperative complications, if any.
3. Performance status (PS) of 0 or 1.
4. The function of major organs is maintained
5. The patient has no serious complications (particularly bowel obstruction, diarrhea or fever).

* For age, see CQ-17.

- For patients who have Stage II colorectal cancer with a high risk of recurrence, the indications for adjuvant chemotherapy are considered (CQ-18).
- For Stage IV resection cases, see CQ-19.

Recommended therapies (CQ-15)

The postoperative adjuvant chemotherapy regimens that were shown to be useful in clinical trials and which are covered by the Japanese National Health Insurance program are as follows:

**Table:** 

Oxaliplatin (OX) combination therapy	CAPOX(Preferred)* FOLFOX (*Preferred*)*
Fluoropyrimidine (FP) monotherapy	Cape 5-FU + *l*-LV UFT + LV S-1

*See CQ-15.

Recommended administration period (CQ-16)

- In principle, the administration period is 6 months.

Comments

① Postoperative adjuvant chemotherapy and treatment regimens are determined with appropriate informed consent, taking into consideration the expected reduction in the risk of recurrence, which is determined based on the tumor characteristics (pathological stage, histological type, primary tumor location, biomarkers), treatment characteristics (adverse events, quality of life, treatment costs, etc.), and patient characteristics (age, comorbidities, preferences for assumed side effects, willingness to undergo treatment). Postoperative adjuvant chemotherapy should be started within approximately 8 weeks after surgery.
② In an integrated analysis of three randomized controlled trials (RCT) for patients with Dukes’ B and Dukes’ C colon cancer, 5-FU + *1*-LV was associated with an increase in overall survival in comparison to surgery alone. In addition, OX combination therapy for patients with Stage III colon cancer was associated with a significant reduction in the risk of recurrence and an improved prognosis in comparison to 5-FU + *1*-LV in 3 RCTs conducted in Europe and the United States [142, 143]. UFT + LV and Cape showed non-inferiority to 5-FU + *1*-LV [144]. S-1 showed non-inferiority to UFT + LV [145]. On the other hand, the non-inferiority of S-1 to Cape was not shown [146] (CQ-15).
③ The administration period of OX combination therapy in postoperative adjuvant chemotherapy for patients with Stage III colon cancer was compared in an integrated analysis of 6 RCTs, including a Japanese RCT (ACHIEVE trial). In the 3-month treatment group, non-inferiority to the 6-month administration group was not shown in any subjects (IDEA collaboration) [147]. However, in the case of CAPOX, the suppression of recurrence in the 3-month administration group was demonstrated to be comparable to that of the 6-month administration group, especially in cases with a low risk of recurrence. In the ACHIEVE trial, the 3-year disease-free survival rates of the 3-month and 6-month administration groups were also similar [148]. The incidence of sensory peripheral neuropathy was significantly lower in the 3-month administration group [149]. On the other hand, with the postoperative adjuvant chemotherapy for colon cancer in Stage IIB/III (TNM-6 edition), 18 months administration of UFT + LV did not demonstrate superiority to six months administration [150]. Furthermore, 12 months administration of Cape for colon cancer in Stage III did not show superiority in the disease-free survival to six months administration [151] (CQ-16).
④ Although there is less evidence to support postoperative adjuvant chemotherapy for rectal cancer than there is for colon cancer, the efficacy is almost the same as that in colon cancer. Thus, it can be carried out with reference to the evidence of colon cancer. In postoperative UFT alone (1 year) for Stage III rectal cancer (including the anal canal), significant suppression of recurrence and a survival benefit in comparison to surgery alone were observed in a Japanese RCT [152]. Thereafter, S-1 (1 year) showed a significant relapse-suppressing effect in comparison to UFT alone (1 year) for Stage II/III rectal cancer (including the anal canal and excluding RS) [153] (CQ-15).
⑤ In an RCT of postoperative adjuvant chemotherapy in Japan for patients with Stage II colon cancer, UFT alone (1 year) did not prevent cancer recurrence in comparison to surgery alone [154] (CQ-18).
⑥ In postoperative adjuvant chemotherapy for patients with Stage II/III colon cancer, the concomitant use of IRI or molecular targeted drugs is not recommended.
⑦ UFT + LV showed significantly better relapse-free survival in comparison to surgery alone in an RCT of adjuvant chemotherapy for patients after curative resection of liver metastasis in Japan [155] (CQ-19).

2. Systemic therapy for unresectable colorectal cancer (Figs. 6 and 7)

**Fig. 6:**
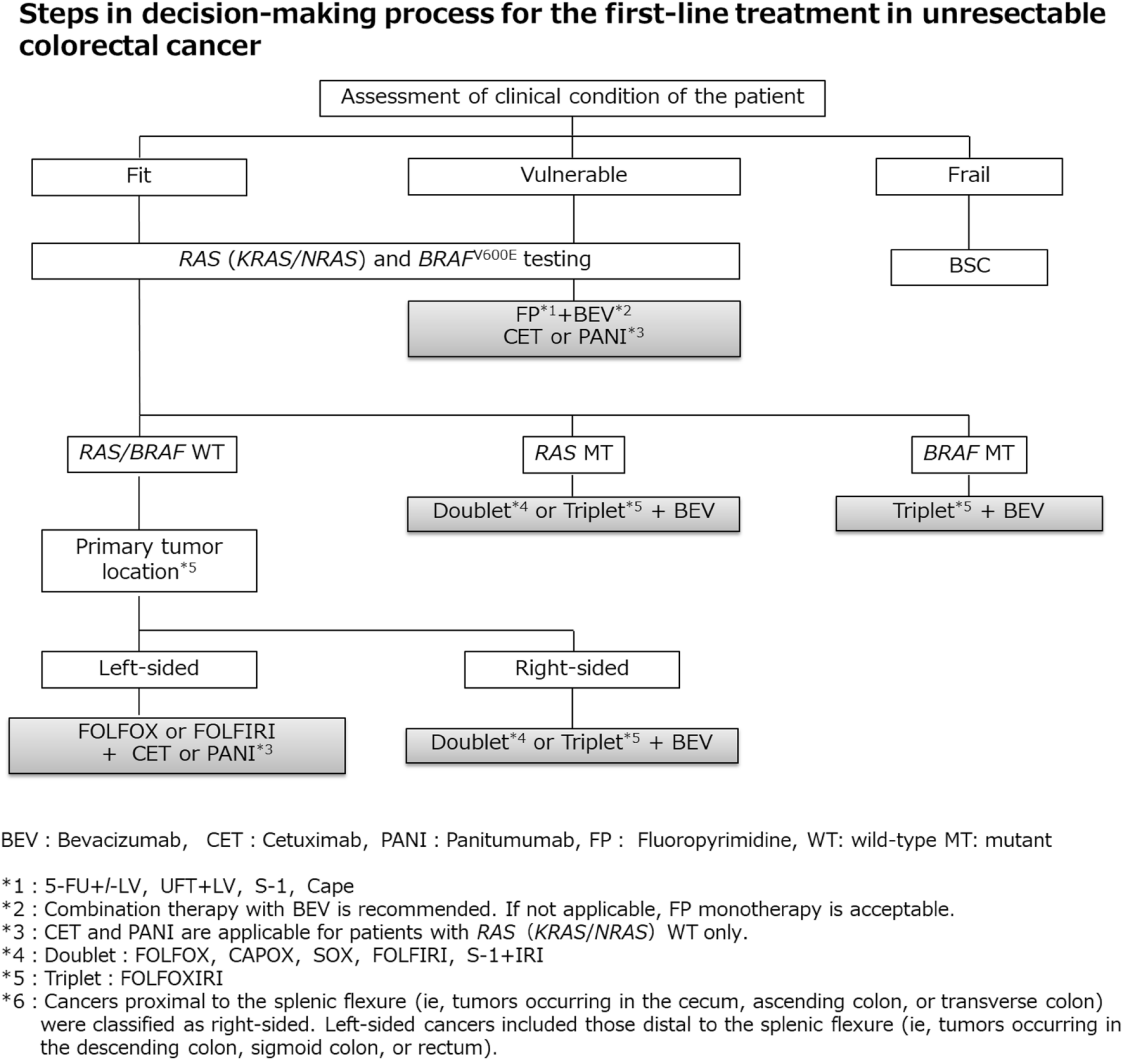
Steps in the decision-making process for the first-line treatment in unresectable colorectal cancer

**Fig. 7:**
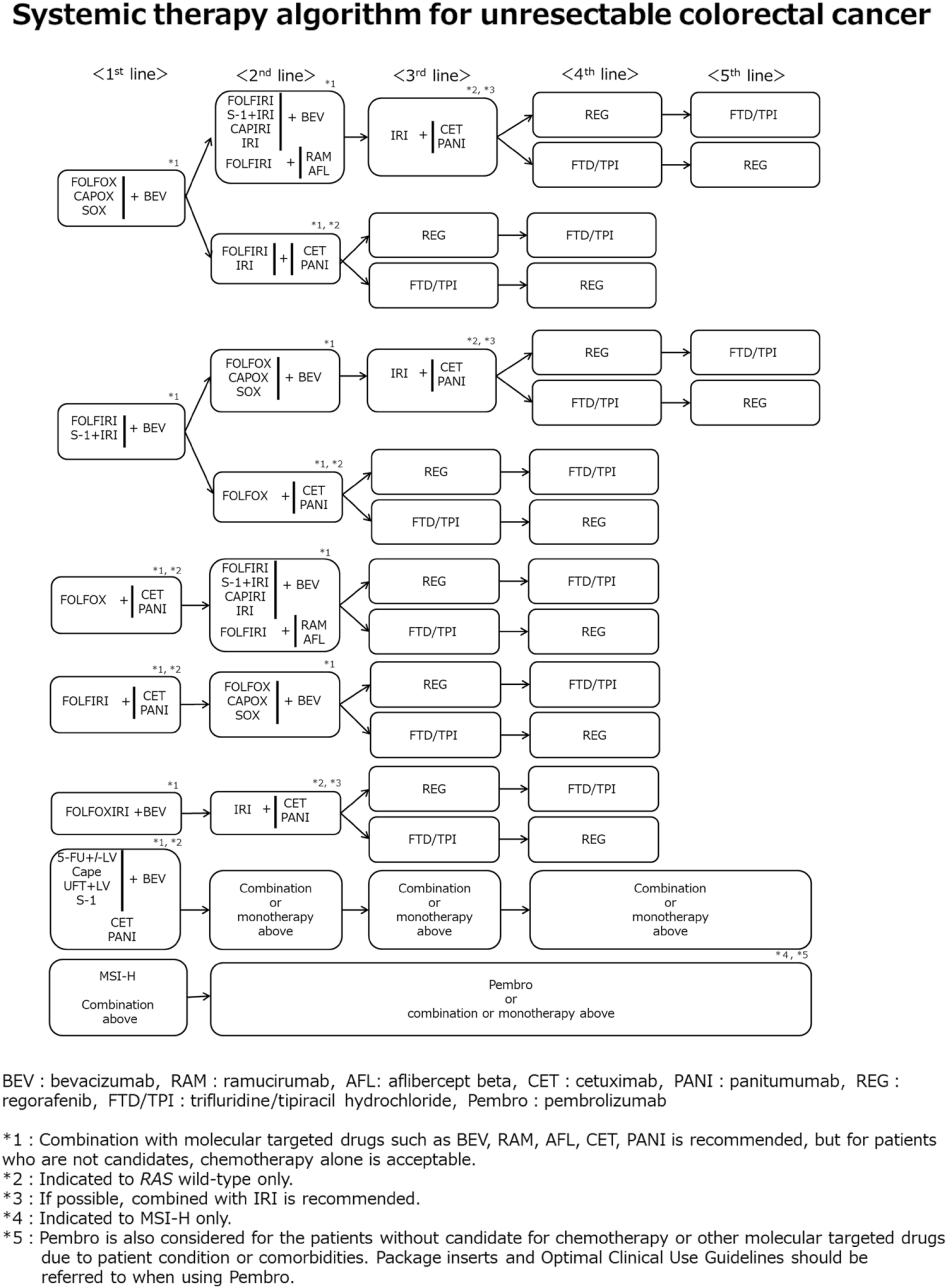
Systemic therapy algorithm for unresectable colorectal cancer

- In the BSC without any systemic therapy, the median survival time (MST) of patients with unresectable colorectal cancer has been reported to be approximately 8 months [156]. Although recent systemic therapy has extended MST to approximately 30 months [157–159], unresectable colorectal cancer remains difficult to cure.
- The purpose of systemic therapy is to prolong survival time and control symptoms by delaying tumor progression. Initially, unresectable colorectal cancer may become resectable after successful systemic therapy.
- Randomized controlled trials involving PS 0–2 patients have shown that systemic therapy is associated with a significantly longer survival time than BSC without any systemic therapy [156, 160].
- When considering systemic therapy, it should first be decided whether or not it is applicable. [The steps in the decision-making process for first-line treatment in unresectable colorectal cancer (Fig. 6)].
- Patients appropriate for intensive systemic therapy (fit) include those with no serious comorbidities who are considered tolerant to first-line therapy with OX and IRI, as well as concomitant therapy with molecular targeted drugs (Fig. 6).
- Patients inappropriate for intensive systemic therapy (vulnerable) include those with some comorbidities who are considered intolerant to first-line therapy with OX and IRI, as well as concomitant therapy with molecular targeted drugs.
- Patients inappropriate for systemic therapy (frail) include those with serious comorbidities who are considered intolerant to first-line therapy.
- *RAS* (*KRAS/NRAS*) mutation testing and *BRAF*^V600E^ mutation testing should be performed before first-line therapy for patients appropriate for systemic therapy.
- CET and PANI are only indicated for patients with wild-type *RAS* (*KRAS/NRAS*) (Comment ⑧ [161]).
- Pembro is only indicated for patients with high-frequency microsatellite instability (MSI-H) (CQ-23).

General principles underlying the indications of systemic therapy

1. The clinical or histopathological diagnosis has been confirmed as colorectal cancer.
2. The curative resection is not possible.
3. Patients are defined as “fit” or “vulnerable” depending on the general condition, the main organ function, and the presence or absence of serious comorbidities (refer to the package insert of each drug).
  The efficacy and safety of the following regimens have been confirmed in clinical trials. These systemic therapies are approved and covered by the Japanese National Health Insurance system.

First-line therapy (CQ-20)

The following regimens are considered as systemic therapy for first-line therapy.

FOLFOX^note 1^ [162, 163] + BEV [164]

CAPOX + BEV [164, 165]

SOX + BEV [157]

FOLFIRI [166, 167] + BEV [159, 168]

S-1 + IRI + BEV (see comment ④) [169]

FOLFOX + CET [170], FOLFOX + PANI [171]

FOLFIRI + CET [172], FOLFIRI + PANI [173]

FOLFOXIRI [174] + BEV [158, 175]

Infusional 5-FU + *l*-LV [176, 177] + BEV [178, 179]

Cape [180, 181] + BEV [182]

UFT + LV [183–185] + BEV [186]

S-1 + BEV [187]

CET or PANI [188, 189]

Second-line therapy (CQ-21)

The following regimens are considered as systemic therapy for second-line therapy.

- For patients who are refractory or intolerant to the first-line therapy, including OX.

FOLFIRI + BEV [190, 191]

CAPIRI + BEV (see comment ⑤) [192]

FOLFIRI + RAM [193]

FOLFIRI + AFL (see comment ⑥) [194]

S-1 + IRI [195] + BEV

IRI [196] + BEV [197]

FOLFIRI (or IRI) + CET, FOLFIRI + PANI [198, 199]

IRI + CET [200] or IRI + PANI [201]

Pembro (see comment ⑩) [202]

(b) For patients who are refractory or intolerant to the first-line therapy, including IRI

FOLFOX [203] + BEV [190, 204]

CAPOX [205] + BEV [190]

SOX + BEV

FOLFOX + CET, FOLFOX + PANI

Pembro (see comment ⑩) [202]

(c) For patients who are refractory or intolerant to the first-line therapy, including 5-FU, OX and IRI

(IRI +) CET [206–208] or (IRI +) PANI [209, 210]

Pembro (see comment ⑩) [202]

Third-line and subsequent therapies (CQ-22)

The following regimens are considered as systemic therapy for third-line and subsequent therapies

(IRI +) CET [206–208] or (IRI) + PANI [209, 210]

REG [211]

FTD/TPI [212–214]

Pembro (see comment ⑩) [202]

Note 1: The efficacy and safety of the underlined regimen have been validated in Phase III trials.

Comments

① When administering OX, it is necessary to pay attention to the cumulative neurotoxicity of OX. Although Grade 2 neurotoxicity which impairs tolerability was observed, if the curative effect persists, stopping OX and switching to fluoropyrimidine ± BEV/CET/PANI, etc., should be considered. If the disease progress and neurotoxicity is improved to Grade 1 or less, reintroduction of OX should be considered.
② Careful attention is required when using IRI for patients with constitutional jaundice, such as that caused by Gilbert`s syndrome, or those with high serum bilirubin or who are in a poor general condition (PS 2). Associations between genetic polymorphisms of enzymes that metabolize IRI (*UGT1A1*) and toxicity have been suggested. Although the maximum tolerated dose of IRI was confirmed to be 150 mg/m2 for patients with *UGT1A1* homozygous (*28/*28, *6/*6, or *28/*6), grade 3 or higher neutropenia was observed in 62.5% of these patients during the first cycle [215].
③ In Japan, the efficacy and safety of FOLFOXIRI + BEV were confirmed in the QUATTRO trial* [216]. This trial enrolled the patients of 20–75 years of age, with PS 0–1 (PS 0: 71–75 years of age), and without *UGT1A1* homozygous (*28/*28, *6/*6, or *28/*6). Thus, the efficacy and safety were not confirmed among other patients. In this study, grade 3/4 neutropenia and febrile neutropenia were observed in 72.5% and 21.7% of patients, respectively. Grade 4 neutropenia and febrile neutropenia during the early cycles were higher in patients heterozygous for *UGT1A1* (*28/*1, *6/*1) in comparison to wild type (*1/*1).
  *The QUATTRO trial, a phase II trial conducted in Japan, confirmed the efficacy and safety of FOLFOXIRI + BEV in first-line therapy for patients with unresectable colorectal cancer.

④ The efficacy and safety of S-1 + IRI + BEV were confirmed in the TRICOLORE trial [169]. The TRICOLORE trial, a phase III trial conducted in Japan, compared the efficacy and safety of S-1 + IRI + BEV with FOLFOX + BEV or CAPOX + BEV as first-line therapy for PS 0–1 patients with unresectable colorectal cancer.
⑤ The efficacy and safety of CAPIRI + BEV were confirmed in the AXEPT trial* [192].
  * The AXEPT trial, a phase III trial conducted in Asia, compared the efficacy and safety of CAPIRI ± BEV with FOLFIRI ± BEV as second-line therapy for PS 0–2 patients with unresectable colorectal cancer.

⑥ The efficacy and safety of AFL were confirmed in the VELOUR trial* [194]. The administration of AFL is approved in combination with 5-FU, *1*-LV, and IRI in Japan. As described in the package insert, the efficacy and safety of AFL in first-line therapy have not been established.
  *The VELOUR trial, an international cooperative phase III trial, compared the efficacy and safety of FOLFIRI + AFL with FOLFIRI + placebo as second-line therapy for PS 0–2 patients with unresectable colorectal cancer who were refractory or intolerant to prior combination therapy with fluoropyrimidine and OX.

⑦ Although hepatic arterial infusion therapy is associated with high response rates in patients with liver metastasis, it does not show any survival benefit in comparison to systemic therapy [217] (CQ-24).
⑧ *RAS* (*KRAS/NRAS*) gene mutations are detected in approximately 50% of patients with unresectable colorectal cancer, and it was reported that the efficacy of anti-EGFR antibody therapy (CET, PANI) cannot be expected for patients with these mutations. Thus, it is recommended that *RAS* (*KRAS/NRAS*) mutation testing should be performed prior to first-line therapy for patients who can receive systemic therapy [218, 219] (CQ-20). In Japan, *RAS* (*KRAS/NRAS*) mutation testing has been reimbursed since April 2015. In a recent pooled analysis that included six randomized trials comparing chemotherapy plus anti-EGFR antibody therapy with chemotherapy or chemotherapy plus BEV, chemotherapy plus anti-EGFR antibody therapy showed superior efficacy in patients with left-sided tumors (descending colon, sigmoid colon, rectum) in comparison to those with right-sided tumors (cecum, ascending colon, transverse colon) [220].
⑨ In Japan, *BRAF*^V600E^ gene mutations are detected in approximately 5% of patients with unresectable colorectal cancer, and patients with these mutations are resistant to systemic therapy and have a very poor prognosis [221, 222]. Based on a subgroup analysis in the TRIBE trial, FOLFOXIRI + BEV therapy might be effective as a first-line therapy for patients with *BRAF*^V600E^ gene mutations [223]. Thus, it is recommended that *BRAF*^V600E^ mutation testing should be performed prior to the administration of first-line therapy for patients who can receive systemic therapy [219, 220] (CQ-20). *BRAF*^V600E^ mutation testing is also useful as an adjunct diagnostic test for Lynch syndrome. Thus, this test is recommended for patients with DNA mismatch repair deficiency and suspected Lynch syndrome. For the basic requirements of the *BRAF*^V600E^ mutation testing, refer to “Japanese Society of Medical Oncology (JSMO) Clinical Guidelines: Molecular testing for Colorectal Cancer Treatment, Third Edition” [220]. In Japan, *BRAF*^V600E^ mutation testing was reimbursed since August 2018. Recently, the efficacy of combination therapy of BRAF inhibitors and anti-EGFR antibodies for patients with *BRAF*^V600E^ gene mutation was reported [224, 225]. In the NCCN guideline version 1.2018, combination therapy of IRI + anti-EGFR antibody + Vemurafenib (BRAF inhibitor) is listed as a recommended regimen in second or later line for these patients (unapproved in Japan as of January 2019).
⑩ MMR (mismatch repair) deficiency was mainly observed in patients with colorectal cancer associated with Lynch syndrome caused by germline mutations of genes associated with MMR or sporadic colorectal cancer caused by acquired *MLH1* gene methylation. Tests for tumor MMR deficiency include MSI testing and immunohistochemistry (IHC) for MMR proteins. According to Western data, MSI-H is recognized in approximately 5% of unresectable colorectal cancer (approximately 2–3% in Japan). There is no established systemic therapy specifically for unresectable colorectal cancer with MMR deficiency. Thus, under the current circumstances, the common regimens for sporadic colorectal cancers are indicated for these patients. Recently, the efficacy of anti-PD-1 antibody therapies (pembrolizumab [Pembro] and nivolumab) against unresectable colorectal cancer with MMR deficiency was reported. In the United States, these therapies are approved for unresectable colorectal cancer with MMR deficiency [202, 226]. In Japan, Pembro was approved for MSI-H solid cancer (only for patients for whom standard systemic therapy is not appropriate), including MSI-H unresectable colorectal cancer, in December 2018 (CQ-23). At the same time, the MSI testing Kit (FALCO) was reimbursed as a companion diagnostic test. MSI testing can also be used in screening for Lynch syndrome. Thus, it is recommended that physicians refer to the “JSCCR Guidelines 2016 for the Clinical Practice of Hereditary Colorectal Cancer” [227] for the explanation of the test, the interpretation of the results, and correspondence in cases of suspected Lynch syndrome. It is reported that the concordance rate between the MSI testing and IHC for MMR protein is high in colorectal cancer.

Chapter 6: Radiotherapy

- Radiotherapy is used to treat patients with locally advanced rectal cancer either as adjuvant therapy after surgery to prevent recurrence or before surgery to reduce tumor volume and preserve the anal sphincter, and also as palliative care to relieve the symptoms and prolong the survival time of patients with unresectable colorectal cancer who have symptomatic lesions.

1. Adjuvant radiotherapy

- Adjuvant radiotherapy is classified into three categories, according to the timing of surgery and radiation therapy: preoperative radiotherapy, intraoperative radiotherapy, and postoperative radiotherapy.
- The purpose of adjuvant radiotherapy is to improve the local control rate and the survival rate of rectal cancer patients. The purpose of preoperative radiotherapy includes improving the anal sphincter preservation rate and improving the resection rate. However, insufficient evidence of improved survival has been found to make this the objective of adjuvant radiotherapy.
- Preoperative radiotherapy is indicated for patients with T stage clinically diagnosed as “invasion depth cT3 or deeper or cN-positive”; postoperative radiotherapy is indicated for patients with T stage pathologically diagnosed after surgery as “invasion depth pT3 or deeper or pN positive, where the existence of a surgical dissection plane positive (RM1) or penetration of the surgical dissection plane by the cancer (RMX) is unclear”; and intraoperative radiotherapy is indicated for “surgical dissection plane positive (RM1) or penetration of the surgical dissection plane by the cancer (RMX) is unclear”.
- Radiotherapy is delivered with a linear accelerator, with electron beams being used for intraoperative radiotherapy and photon beams for external radiotherapy.

Comments

① Preoperative radiotherapy (CQ-25)
  1. Preoperative radiotherapy has the following advantages: seeding during surgery can be prevented by inactivating lesions with irradiation; a high percentage of tumor cells are normo-oxic and radiosensitive, because blood flow to the tumor is maintained; there has been little damage to the digestive tract, since the small bowel is not fixed within the pelvic cavity, thereby resulting in low radiation-induced delayed toxicity, which means a less toxic postoperative setting; improvement in the R0 resection rate and anal sphincter preservation can be expected because of tumor size reduction [228].
  2. Preoperative radiotherapy has the following disadvantages: early-stage patients may be subjected to overtreatment and postoperative complications may increase.
  3. Twelve phase III clinical trials of preoperative radiotherapy (without chemotherapy) have been reported [228], and in 5 of the 12 randomized controlled trials the local control rate in the group that received preoperative radiotherapy was significantly higher than in the surgery-alone group. However, an improvement in the survival rate was observed in only 1 trial [229].
  4. Two meta-analyses of radiotherapy showed improvement in the local control rate compared to surgery alone, and improvement in the survival rate in the groups that received doses of 30 Gy or more. However, there is controversy as to whether there is improvement in the survival rate [230, 231].
  5. Trials of short-course radiotherapy with 5 Gy per fraction have been conducted, mainly in Europe [229, 232]. Because the late effects of radiation depend on the fraction size, long-term follow-up for late adverse effects, such as anal dysfunction and bowel dysfunction, is necessary.
  6. In the Dutch CKVO 95-04 trial, which compared preoperative radiotherapy (25 Gy delivered in five fractions in one week) + TME and TME alone to investigate the significance of adding short-course radiotherapy to TME, the 5-year and 10-year local control rates were significantly higher in the combination therapy group, but there was no significant difference between the two groups in the 5-year and 10-year survival rates [138, 232, 233]. The incidences of sexual dysfunction and bowel dysfunction were higher in the preoperative radiation combination therapy group than in the surgery-alone group [234, 235].
  7. The effect of preoperative radiotherapy in reducing the size of the primary tumor may enable sphincter preservation. When the purpose of the preoperative radiotherapy is sphincter preservation, it is desirable to perform surgery after allowing an appropriate period for the tumor to decrease in size (6–8 weeks after the completion of radiotherapy) [236].
  8. In Europe, four randomized controlled trials, including the EORTC trial, were performed to investigate the usefulness of adding chemotherapy to preoperative radiotherapy. The incidence of acute-phase adverse events was significantly higher in the preoperative chemoradiotherapy groups, but the pathologic complete response rates (pCR) were significantly higher than that in the preoperative radiotherapy alone groups. In two trials, the exception being the short-course radiotherapy trial, the local recurrence rate was significantly lower in the preoperative chemoradiotherapy group, and there was no significant difference between the two groups in terms of sphincter preservation or survival rate [237–240].
  9. In a randomized controlled trial that compared preoperative chemoradiotherapy and postoperative chemoradiotherapy, there was no significant difference in the 5-year survival rate, but the local recurrence rate and incidence of grade 3 or higher adverse events were significantly lower in the preoperative chemoradiotherapy group. Among the patients in whom abdominoperineal resection (APR) was considered necessary at the time of enrollment, the percentage of patients in whom sphincter preservation was possible was significantly higher in the preoperative chemoradiotherapy group [241].
  10. A randomized controlled trial of 5-FU versus Cape combination chemotherapy in the preoperative chemoradiotherapy indicated that the two drugs had the same level of efficacy and safety [242, 243]. NCCN guidelines allow the use of either 5-FU or Cape as standard combination chemotherapy in the preoperative chemoradiotherapy. The indications and use of Cape as an adjuvant therapy for rectal cancer have been approved for use under health insurance in Japan as of August 2016.
  11. In randomized controlled trials into the efficacy of adding OX to fluoropyrimidine as a combination chemotherapy in the preoperative chemoradiotherapy, OX increased adverse events in three trials, but demonstrated no efficacy with regard to pCR ratio, localized control ratio and survival [242, 244–246]; moreover, in one trial, although there was no difference in adverse events and no analysis was done into disease-free survival at the primary endpoint, the pCR ratio was significantly higher [247].

2. Palliative radiotherapy
  - Intrapelvic lesions (CQ-26)
    The purpose of palliative radiotherapy for intrapelvic lesions is to relieve symptoms such as pain, hemorrhage, and bowel dysfunction caused by intrapelvic tumors.
    The target volume includes the tumor causing the symptoms.
    [Dose and fractionation]
    A total dose of 45–50 Gy is administered in 1.8–2.0 Gy fractions.
    Depending on the patient’s general condition, such as performance status, and the severity of the symptoms, radiotherapy may be completed in a shorter term with a larger fraction size, for example 30 Gy in 10 fractions over 2 weeks.

(b) Extrapelvic lesions
  1. Bone metastases
    The purpose of palliative radiotherapy for bone metastases is to achieve pain relief, prevent pathological fractures, and prevent and treat spinal cord paralysis.
    The target volume includes the metastatic bone lesions causing the symptoms.
    [Dose and fractionation]
    Local field radiotherapy, such as 30 Gy in 10 fractions and 20 Gy in 5 fractions, is widely performed.
  2. Brain metastases
    See the section on hematogenous metastases (Chapter 4).
    [Dose and fractionation]
    When whole-brain radiotherapy is performed, 30 Gy in 10 fractions is the standard treatment. If long-term survival is expected, fractionated radiotherapy, such as 37.5 Gy in 15 fractions and 40 Gy in 20 fractions, is considered.
    When stereotactic radiosurgery is performed, a peripheral dose of 16–25 Gy is delivered in a single fraction.

Chapter 7: Palliative care

- Palliative care is a general term for palliative treatment of various mental and physical symptoms related to cancer.
- Palliative care extends from the time the diagnosis of disease is made to the end stage, and different care should be provided depending on the disease stage and symptoms.
- In principle, cancer treatment should be performed under conditions in which symptom relief is achieved [248], and palliative care should be started at the same time as surgical treatment and systemic therapy.
- Palliative care to improve the QOL of patients with end-stage colorectal cancer includes:
  1. Pain relief
  2. Surgical treatment
  3. Systemic therapy
  4. Radiotherapy
  5. Counseling for psychiatric symptoms

Chapter 8: Surveillance after surgery for colorectal cancer

1. Surveillance for recurrence after curability A resection of colorectal cancer
2. Consideration should be given to periodic endoscopic examination for recurrence at the site of local resection or anastomosis in pStage 0 [pTis cancer] cases. Surveillance for recurrence in other organs is not necessary.
3. pStage I–pStage III cases should be surveyed for recurrence in the liver, lungs, local area, anastomosis, lymph nodes, peritoneum, etc. The following points should be noted.
  In principle, the duration of surveillance is 5 years after surgery, and the surveillance examinations should be scheduled at shorter intervals during the first 3 years after surgery.
  It should be noted that there is a higher incidence of lung metastasis and local recurrence in rectal cancer than in colon cancer.
  The following is an example of a surveillance schedule after curative resection of Stage I to Stage III colorectal cancer that was designed on the basis of the results of a retrospective investigation of such factors as the common sites and incidence of recurrence, the efficacy of treatment, and the clinical practice in Japan. (Figure 8)

**Fig. 8:**
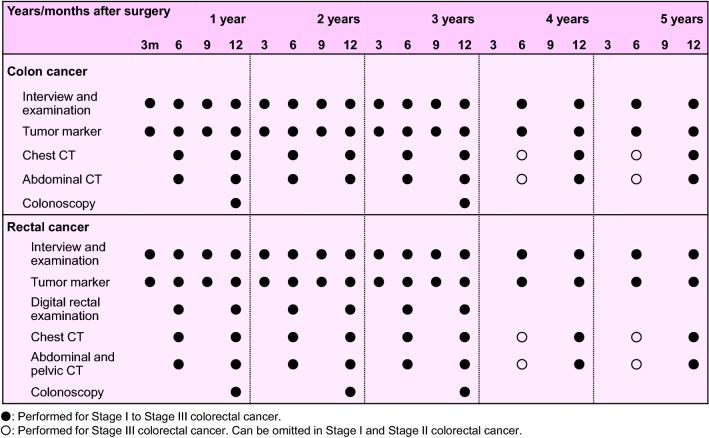
An example of a surveillance schedule after curative resection of pStage I to pStage III colorectal cancer
4. Surveillance after curability B resection of colorectal cancer and after resection of recurrent tumors.

1. The same surveillance method as for Stage III colorectal cancer is used. It should be noted that recurrence and re-recurrence are common in organs previously operated on. It should also be noted that the frequency of relapse after 5 years is relatively high.
2. In cases allocated curability B due to R1 resection, close surveillance schedule should be planned for organs in which residual cancer is suspected.

3. Surveillance of metachronous multiple cancer
  Colonoscopy is performed for surveillance of metachronous multiple colorectal cancer.

Comments

① Aim of surveillance
  The aim of surveillance is to improve the patient’s prognosis by early detection and treatment of recurrences [249]. Thus, surveillance is conducted for patients who can be treated when recurrence is found [250].
② Recurrence rate, sites of recurrence, times of recurrence
  The results of the JSCCR colorectal cancer registry in 2007 are shown in Figs. 9 and 10 and Tables 10, 11, 12, and 13. The subjects included 5,103 patients who underwent curative resection of colorectal cancer in 2007 at the 71 institutions that participated in the registry. The median follow-up period was 6.0 years.

**Fig. 9:**
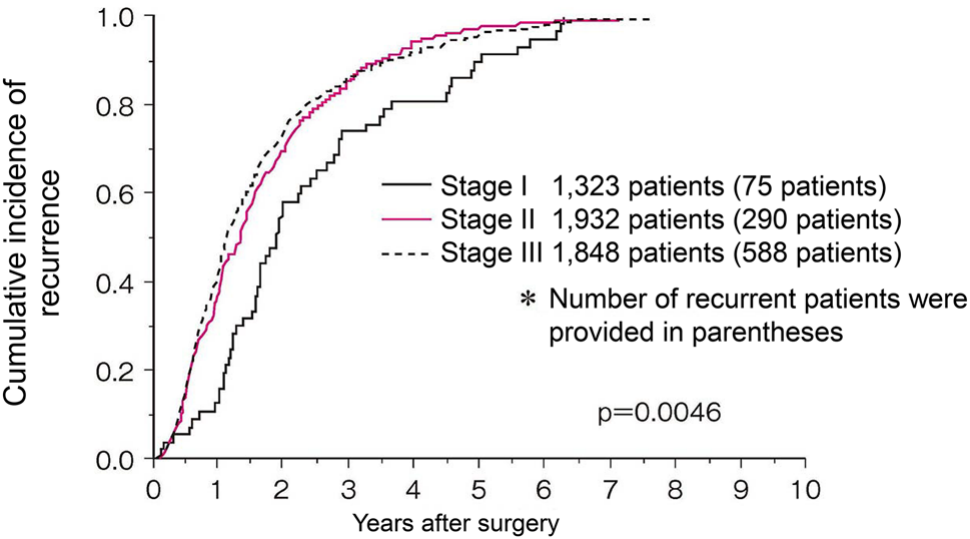
Cumulative incidence of recurrence according to stage (JSCCR colorectal cancer registry: patients in the year 2007)

**Fig. 10:**
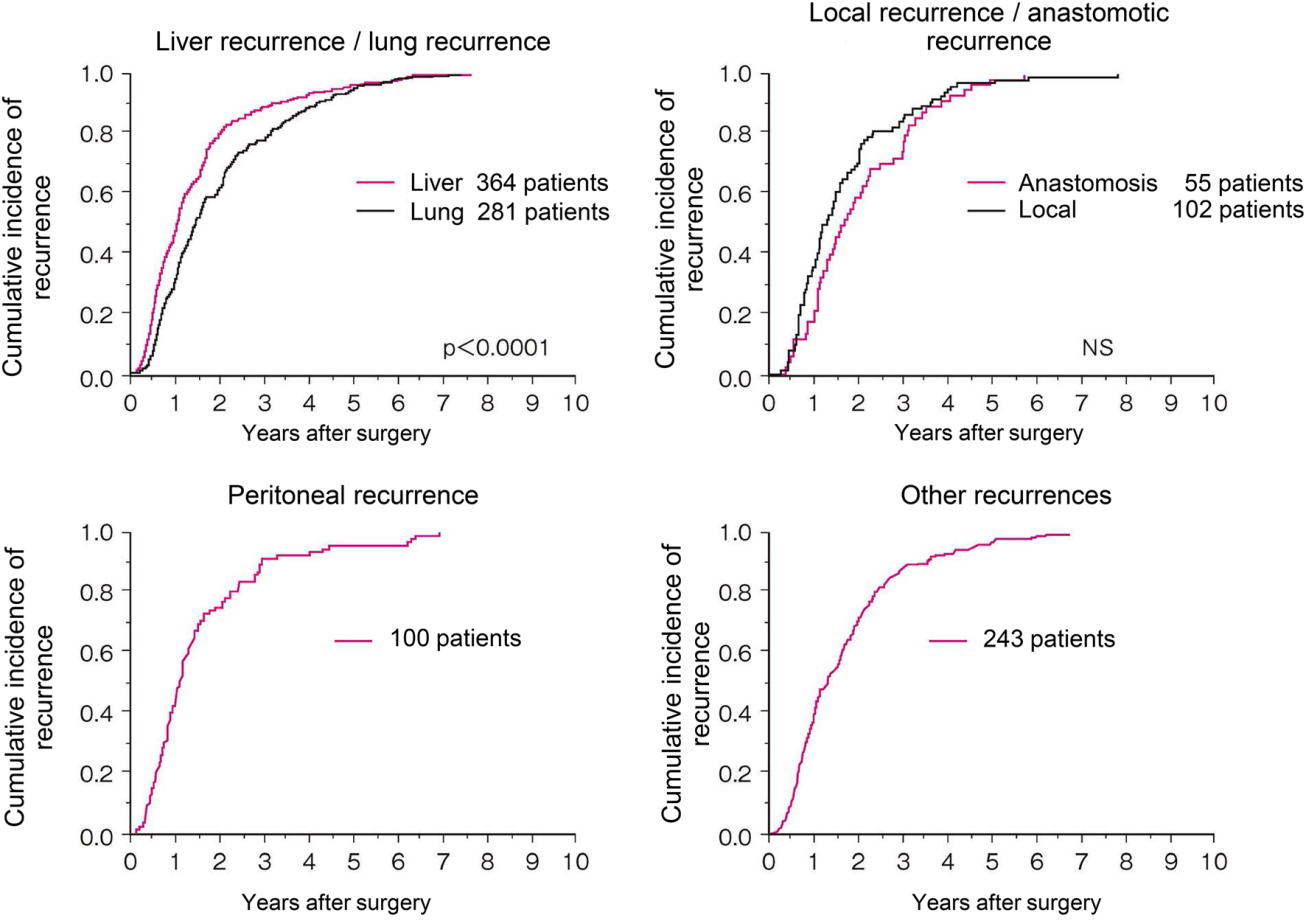
Cumulative incidence of recurrence according to the site of recurrence (JSCCR colorectal cancer registry: patients in the year 2007)

**Table 10:** Recurrence rate after curative resection of colorectal cancer according to pStage and cumulative incidence of recurrence according to the number of years after surgery

pStage (no. of patients)	Recurrence rate (no. of patients with recurrence)	Cumulative incidence of recurrence according to the no. of years after surgery (cumulative no. of patients with recurrence)			Percentage of patients experiencing recurrence more than 5 years after surgery among all patients (no. of patients)
		3 years	4 years	5 years	
I	5.7%	73.7%	80.7%	91.2%	0.4%
(1323)	(75)	(42)	(46)	(52)	(5)
II	15.0%	86.0%	94.2%	97.7%	0.3%
(1932)	(290)	(221)	(242)	(251)	(6)
III	31.8%	86.7%	92.0%	96.5%	1.1%
(1848)	(588)	(475)	(504)	(529)	(19)
All	18.7%	85.6%	91.9%	96.5%	0.6%
(5103)	(953)	(738)	(792)	(832)	(30)

JSCCR colorectal cancer registry (patients in the year 2007); 91 patients were excluded from analyses for cumulative incidence of recurrence because of unknown recurrence date

**Table 11:** Recurrence rate of pStage I colorectal cancer

pStage I	No. of patients	No. of patients with recurrence	Recurrence rate (%)	*p* value
Tumor location				
Colon	756	33	4.4	*p* = 0.0186
Rectum	567	42	7.4	
Depth of tumor invasion				
SM	655	26	4.0	*p* = 0.0076
MP	668	49	7.3	
Tumor location and depth of tumor invasion				
Colon				
SM	403	10	2.5	*p* = 0.0063
MP	353	23	6.5	
Rectum				
SM	252	16	6.4	NS
MP	315	26	8.3	

JSCCR colorectal cancer registry: (patients in the year 2007); RS cancer was counted as rectal cancer

**Table 12:** Recurrence rate according to the site of the first recurrence after curative resection of colorectal cancer and cumulative incidence of recurrence according to the number of years after surgery

Site of first recurrence	Recurrence rate (no. of patients with recurrence (including overlaps)	Cumulative incidence of recurrence according to the number of years after surgery (cumulative No. of patients with recurrence)			Percentage of patients experiencing recurrence more than 5 years after surgery among all patients (no. of patients)
		3 years	4 years	5 years	
Liver	7.1%	89.3%	93.8%	96.4%	0.24%
	(364)	(301)	(316)	(325)	(12)
Lung	5.5%	79.2%	89.2%	95.8%	0.22%
	(281)	(206)	(232)	(249)	(11)
Peritoneum	2.0%	91.3%	93.5%	95.7%	0.09%
	(100)	(84)	(86)	(88)	(4)
Local	2.0%	86.0%	95.7%	97.9%	0.04%
	(102)	(80)	(89)	(91)	(2)
Anastomotic	1.1%	81.1%	92.5%	98.1%	0.02%
	(55)	(43)	(49)	(52)	(1)
Other	4.8%	89.6%	93.2%	98.2%	0.08%
	(243)	(198)	(206)	(217)	(4)
All	18.7%				
(5103)	(953)				

JSCCR colorectal cancer registry: (patients in the year 2007); 91 patients were excluded from analyses for cumulative incidence of recurrence because of unknown recurrence date

**Table 13:** Comparison of the recurrence rates between colon cancer and rectal cancer according to the site of the first recurrence

Site of recurrence	Colon cancer (3135 patients)	Rectal cancer (1968 patients)	*p* value
Liver	7.2% (227)	7.0% (137)	NS
Lung	3.9% (121)	8.1% (160)	*p* < 0.0001
Peritoneum	2.5% (79)	1.1% (21)	*p* = 0.0001
Local	0.7% (22)	4.1% (80)	*p* < 0.0001
Anastomotic	1.0% (30)	1.3% (25)	NS
Other	4.0% (124)	6.1% (119)	*p* = 0.0007
All	16.0 (502)	22.9% (451)	*p* < 0.0001

JSCCR colorectal cancer registry: (patients in the year 2007); RS cancer was counted as rectal cancer
(1) Timing of recurrence and sites of the recurrences (Figs. 9, 10, Tables 10, 12, 13).
  More than 85% of the recurrences were detected within 3 years after surgery, and more than 95% of the recurrences were detected within 5 years after surgery.
  The overall incidence of recurrence more than 5 years after surgery was less than 1%.
  The appearance of pulmonary recurrence tended to be slower than that of liver metastasis.
  Local recurrence and lung recurrence were more frequent in rectal cancer than in colon cancer. In contrast, peritoneal recurrence was more frequent in colon cancer than in rectal cancer.
(2) Characteristics of recurrence according to pStage (Fig. 9, Tables 10, 11)
  1. pStage I
    The recurrence rates of colon cancer and rectal cancer were 4.4% and 7.4%, respectively. Rectal cancer was associated with a higher rate of recurrence.
    The recurrence rates of pT1 cancer and pT2 cancer were 4.0%, and 7.3%, respectively.
    In pStage I cases, the appearance of recurrence was delayed in comparison to pStage II and pStage III cases, and recurrence appeared after 5 years in more than 8% of patients with recurrent disease. Among all pStage I cases, the proportion of patients with recurrence after 5 years was less than 0.5%.
2. pStage II, pStage III
  The recurrence rates of pStage II and pStage III were 15.0% and 31.8%, respectively.
  Recurrence was detected within 3 years after surgery in more than 85% of patients with recurrent disease.
  The incidence of recurrence at more than 5 years after surgery in patients with pStage II and pStage III colorectal cancer was 0.3% and 1.1%, respectively.
③ Surveillance for recurrence after curability A resection of colorectal cancer
  The surveillance schedule shown in Fig. 8 was prepared in consideration of the frequency of recurrence for each stage, the site and timing of recurrence, and current surveillance practices in Japan.
  The diagnostic modalities and schedule density differ between the guidelines. The current surveillance methods in Japan are generally intensive in comparison to those adopted in representative guidelines from Western countries (NCCN [251], ESMO [252], ASCO [250, 253], ASCRS [254].
④ Surveillance of metachronous multiple primary cancers
  A past history of colorectal cancer, regardless of stage, is a risk factor for metachronous colorectal cancer [255].
  The recommended interval between colonoscopy ranged from 1 to 5 years, depending on the report [256].
  The need for surveillance targeting multiple primary cancers should be determined by distinguishing hereditary colorectal cancer [257]. There is little evidence of a need for periodic minute examinations for cancer in other organs following surgery for sporadic colorectal cancer (CQ-28).

Clinical questions

CQ-1: What are the indication criteria for additional treatment after endoscopic resection of pT1 colorectal cancer? (Fig. 11)

**Fig. 11:**
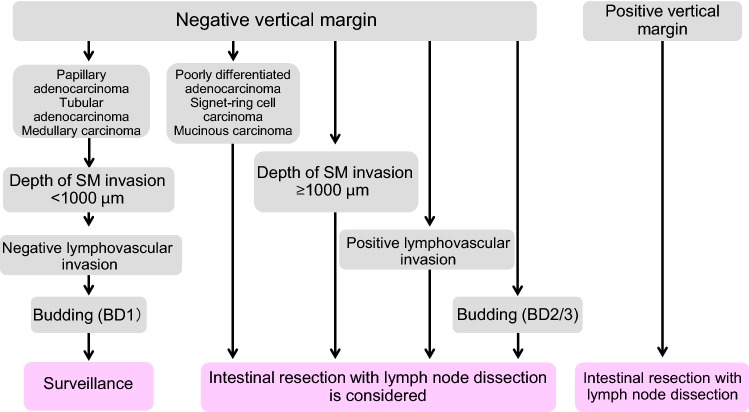
Treatment strategies for pT1 cancer after endoscopic resection

① Surgical resection is recommended when the vertical margin is positive (Recommendation 1/Evidence level C)
② If any of the following findings is observed during histological examination of the resected specimen, intestinal resection with lymph node dissection is recommended as an additional treatment (Recommendation 2/Evidence level B)
  1. T1b (depth of SM invasion ≥ 1000 µm)
  2. Lymphovascular invasion positive
  3. Poorly differentiated adenocarcinoma, signet-ring cell carcinoma, or mucinous carcinoma [258]
  4. Budding grade of BD2/3 at the site of deepest invasion [258]

Note

- “Positive vertical margin” means that carcinoma is exposed at the submucosal margin of the resected specimen.
- Depth of SM invasion and the grade of budding are measured by the method described in “Japanese Classification of Colorectal, Appendiceal, and Anal Carcinoma, third English edition” [2].

In cases with a positive vertical margin, the recurrence risk is estimated to be higher if follow-up is carried out in comparison to those with a negative vertical margin, since local remnant cancer is a matter of great concern. Furthermore, it is difficult to make an accurate pathological evaluation of the invasive front of the tumor on resected specimens. Although the evidence level is C, considering the balance between harm and benefit, we decided on a “strong recommendation” based on the result of the vote of the committee.

The principle for treatment of pT1 carcinomas, which are invasive carcinomas, is intestinal resection with lymph node dissection. However, some pT1 (SM) carcinomas have a very low risk of metastasis, and the purpose of these criteria is to minimize the need for additional resections that eventually result in overtreatment of such patients. While no diagnostic methods make it possible to predict lymph node metastasis (pN) without fail, the degree of risk of metastasis can be used as a basis for determining whether or not to perform additional treatment.

Factors such as the depth of submucosal invasion (SM invasion depth) [259], histological type, such as poorly differentiated adenocarcinoma, signet-ring cell carcinoma, and mucinous carcinoma [256], the presence of a poorly differentiated area and muconodules at the site of deepest invasion, budding, and lymphovascular invasion, have been reported to be risk factors for regional lymph node metastasis by pT1 (SM) carcinoma [258, 260].

The above criteria for determining whether additional treatment is indicated were prepared based on the following three criteria for performing additional intestinal resection of pT1 (SM) carcinoma described in the “Japanese Classification of Colorectal Carcinoma” (2nd edition, 1980): [(1) Obvious intravascular carcinoma invasion; (2) Poorly differentiated adenocarcinoma or undifferentiated carcinoma; (3) Massive carcinoma invasion extending to the vicinity of the margin] [261]. The description of “Massive carcinoma invasion” in the 4th edition of the “Japanese Classification of Colorectal Carcinoma” was revised to the following more specific description in the 5th edition (1994): “Invasion deeper than ‘very shallow invasion’ (e.g., invasion exceeding approximately 200–300 µm)” [262].

Subsequent case series studies in Japan have shown that “200 µm to 300 µm” can be extended to 1000 µm [263]. According to the results of the project study by the JSCCR, the lymph node metastasis rate of colorectal carcinoma with an SM invasion depth of 1000 µm or more was 12.5% (Table 14) [256, 263]. However, not all cases with submucosal invasion deeper than 1,000 µm necessarily require additional surgery. Approximately, 90% of patients with a depth of invasion of 1000 µm or more did not have lymph node metastasis, and it is important to determine whether additional treatment is indicated after sufficiently considering other factors in addition to depth of SM invasion, such as whether other risk factors for lymph node metastasis are present, the physical and social background of the patient, and the patient’s wishes.

**Table 14:** Depth of invasion of sm cancer and lymph node metastasis (modified from Ref. [259])

sm invasion distance (μm)	Pedunculated		Non-pedunculated	
	Number of lesions	*n* (+) (%)	Number of lesions	*n* (+) (%)
head invasion	53	3 (5.7)		
0 < *X *< 500	10	0 (0)	65	0 (0)
500 ≤ *X* < 1000	7	0 (0)	58	0 (0)
1000 ≤ *X* < 1500	11	1 (9.1)	52	6 (11.5)
1500 ≤ *X* < 2000	7	1 (14.3)	82	10 (12.2)
2000 ≤ *X* < 2500	10	1 (10.0)	84	13 (15.5)
2500 ≤ *X* < 3000	4	0 (0)	71	8 (11.3)
3000 ≤ *X* < 3500	9	2 (22.2)	72	5 (6.9)
3500 ≤ *X*	30	2 (6.7)	240	35 (14.6)

The lymph node metastasis rate of patients with a depth of invasion of 1000 μm or above was 12.5%

All 3 lymph node metastasis-positive patients with head invasion were lymphatic invasion positive (Ly1)

It has been reported that the incidence of lymph node metastasis is 1.3% (95% confidence interval 0–2.4%) in cases with an SM invasion degree of 1,000 µm or more without risk factors for lymph node metastasis (other than the degree of SM invasion). However, in the event of metastasis or recurrence, a salvage operation cannot be indicated in many cases and cancer death may occur. These risks should be sufficiently discussed among the medical staff, including surgeons.

We added budding as a factor for considering additional treatment in the 2009 edition [264]. Furthermore, project research is currently underway into other histopathological factors. Multicenter joint research projects have produced reports providing the results of consideration into the appropriateness of these criteria [32, 265–267]. Regarding the criteria overseas, the European Society of Gastrointestinal Endoscopy (ESGE) Guideline recommends surgery when lymphovascular invasion, infiltration deeper than 1,000 µm, positive/nonevaluable vertical margins, or poorly differentiated tumor with submucosal invasion are diagnosed referring to JSGE guidelines [268].

CQ-2: Is endoscopic submucosal dissection (ESD) recommended for lesions with a maximum diameter of 2 cm or more?

- Endoscopic resection for lesions with a maximum diameter of 2 cm or more includes EMR, piecemeal EMR, and ESD [33, 269–272].

An accurate preoperative endoscopic diagnosis is essential in endoscopic resection. Selection of EMR, piecemeal EMR, or ESD is determined after taking the operator’s skill into consideration.

As a general rule, en bloc resection is recommended for suspected cancer lesions. If en bloc EMR is judged to be difficult, we recommend ESD (en bloc resection) by a skillful endoscopist.

(Recommendation 1/Evidence level B)

CQ-3: Is surveillance recommended after endoscopic resection of early colorectal cancer?

① When en bloc endoscopic resection is completed with a negative margin, then it is recommended that surveillance should be performed by endoscopic examination for approximately 1 year for the purpose of searching for any metachronous colon tumors (Recommendation 2/Evidence level B)
② When piecemeal endoscopic resection is conducted with a positive horizontal margin, then it is recommended that surveillance should be performed by endoscopic examination for approximately 6 months, as the risks for local recurrence are increased (Recommendation 1/Evidence level C)
③ When an additional intestinal resection is not carried out for pT1 cancer, it is recommended that surveillance should be performed via endoscopic examination along with image diagnoses such as CT and tumor markers for the purpose of searching for lymph node metastasis and distant metastasis (Recommendation 1/Evidence level B)

CQ-4: Is laparoscopic surgery recommended for colorectal cancer?

- Laparoscopic surgery is recommended as an option for colorectal cancer surgery (Recommendation 2/Evidence level B).

However, the patient should be instructed that the efficacy of laparoscopic surgery for transverse colon cancer and rectal cancer is not well established.

The difficulty for locally advanced cancer and patients with obesity and adhesion is high, so the indications should be determined while taking into consideration the skill of each surgical team.

CQ-5: Is lateral lymph node dissection recommended for rectal cancer?

Lateral lymph node dissection is indicated when the lower border of the tumor is located distal to the peritoneal reflection and the tumor has invaded beyond the muscularis propria. The diagnostic criteria for lateral lymph node metastasis have not been established. At present, the criteria for cases where lateral lymph node dissection can be omitted are not clear.

① It is recommended that lateral lymph node dissection should be performed if a preoperative or intraoperative diagnosis reveals the presence of lateral lymph node metastasis (Recommendation 1/Evidence level C)
② Lateral lymph node dissection is recommended, even if lateral lymph node metastasis is not detected by a preoperative or intraoperative diagnosis. Although the survival benefit of lateral lymph node dissection in this group of patients is limited, it can be expected to suppress local recurrence (Recommendation 2/Evidence level B)

Comments

According to retrospective studies in Japan, lateral lymph node metastasis exists in 16–23% of cases of lower rectal cancer (Table 6) [40, 273–276]. Although the prognosis of these cases is poor, in general, 40–50% of patients with R0 resection reportedly achieved five-year survival [40, 274, 276–280]. The efficacy of lateral lymph node dissection is particularly high for patients with lateral nodal involvement in whom the number of lymph node metastases or the number of involved lateral lymph node station is limited [281, 282]. A propensity score matching analysis of pT3/T4 lower rectal cancer cases in the 1995–2004 JSCCR colorectal cancer registry also showed that the 5-year overall survival rate of patients with lateral lymph node dissection was better than that of those without dissection (68.9% vs. 62.0%) [283]. It is considered that there is a high likelihood of achieving a survival improvement by lateral lymph node dissection. Although the evidence level is C, considering the balance between harm and benefit, this was made a “strong recommendation” based on the result of a vote by the committee.

It has been reported that the incidence of lateral lymph node metastasis remains high after preoperative chemoradiation therapy if the lateral lymph nodes are enlarged before treatment. Thus, even in cases in which preoperative chemoradiotherapy is performed, the omission of lateral lymph node dissection is not recommended [284, 285].

Regarding the clinical value of lateral lymph node dissection in cases without obvious lateral lymph node metastasis, the JCOG0212 study examined the non-inferiority of the mesorectal excision (ME) alone to the mesorectal excision with lateral lymph node dissection (ME + LLND) with the primary endpoints of relapse-free survival. This study was conducted for patients with no lateral lymph nodes with a short-axis diameter of 10 mm or more on preoperative CT or MRI and whose tumor was located in the rectum, with the lower tumor margin below the peritoneal reflection. As a result, the non-inferiority of ME alone to ME + LLND was not statistically proven (P value for non-inferiority = 0.0547) [286]. The frequency of local recurrence in the ME + LLND group was significantly lower than that in the ME alone group (7.4% vs. 12.6%). On the other hand, the relapse-free survival curves of the two groups were very similar, and there was no significant difference in either the overall survival rate or local recurrence-free survival rate as a secondary endpoint. Thus, the survival benefit of lateral lymph node dissection was limited in cases without lateral lymph node enlargement. Taken together, the omission of lateral lymph node dissection is not uniformly recommended, even for cases without the enlargement of lateral pelvic lymph nodes, from the viewpoint of local control. The application of lateral lymph node dissection should be determined in individual patients by comprehensively considering the balance between the expected benefits in terms of local control and survival improvement and the surgical risk and postoperative dysfunction.

CQ-6: Is resection of the primary tumor recommended for patients with unresectable distant metastases?

- If symptoms exist as a result of the primary tumor, which are difficult to control using other therapies, and the resection is not significantly invasive, primary tumor resection and early systemic therapy are recommended (Recommendation 1/Evidence level C)

For cases in which no symptoms are caused by the primary tumor, however, the efficacy of resecting the primary tumor has not been established.

CQ-7: In cases where peritoneal metastasis is noted, is the resection of peritoneal metastasis at the same time as the primary lesion recommended?

- If the metastasis is localized (P1, P2) and the resection is not significantly invasive, then the peritoneal metastasis should be resected at the same time as the primary tumor (Recommendation 1/Evidence level C)

Comments

Some cases of long-term survival have been reported in which localized peritoneal metastasis (P1, P2) was resected alongside the primary tumor [287–290].

Simultaneous localized dissemination (P1, P2) that can be excised without excessive risk should be resected along with the primary tumor. It should be noted that it is more effective to excise localized dissemination (P1, P2) without hematogenous metastasis together with the primary tumor [291, 292]. It is considered that improved survival can be highly expected. Thus, it was decided that this should be a “strong recommendation,” although the evidence level is C.

CQ-8: Is resection recommended for cases in which metastases are simultaneously noted in the liver and lungs?

- The efficacy of resection in patients who have liver and lung metastases at the same time has been shown and, thus, resection should be considered for patients with resectable liver and lung metastases (Recommendation 2/Evidence level D)

CQ-9: Is neoadjuvant chemotherapy recommended for cases with resectable liver metastasis?

- The efficacy and safety of neoadjuvant chemotherapy for resectable liver metastasis have not been established (No recommendation/Evidence level C)

CQ-10: Is resection of liver/lung metastasis recommended, if it becomes possible as a result of the effects of chemotherapy?

- Resection should be performed for cases in which chemotherapy has successfully made localized metastasis to the liver or lungs operable (Recommendation 2/Evidence level C)

CQ-11: Is resection of liver metastasis recommended, if it becomes invisible as a result of the effects of chemotherapy?

- Resection is recommended if liver metastasis is no longer visible on both CT and MRI after chemotherapy (Recommendation 2/Evidence level D)

Comments

Approximately, 20–25% of metastatic liver lesions have been reported to disappear after 6–12 courses of medication. However, even if a complete response is observed on imaging, a pathological complete response (disappearance of tumor cells) is not always obtained [293]. There is a high possibility that tumor cells will remain and it is, therefore, recommended that site of the disappearing liver metastasis be excised [294–297].

CQ-12: Is laparoscopic surgery recommended for liver metastasis of colorectal cancer?

- If a well-experienced surgical team carefully considers adaptation, the safety of laparoscopic hepatectomy for colorectal cancer liver metastasis has been confirmed to be nearly equivalent to that of laparotomy. However, with respect to efficacy, the evidence is insufficient and it is not a standard surgical procedure for liver metastasis of colorectal cancer (No recommendation/Evidence level D)

CQ-13: Is thermal ablation therapy recommended for metastatic liver lesions?

There are few reports indicating the efficacy of thermal ablation therapy. Since thermal ablation therapy is accompanied by a high risk of local recurrence in cases of liver metastasis, resection should be initially considered wherever possible.

① As resection is the standard therapy for resectable lesions, it is not recommended as the first choice of treatment (Recommendation 1/Evidence level C)
② As systemic therapy is the standard therapy for unresectable liver metastasis, it is not recommended for unresectable lesions (Recommendation 2/Evidence level C)

CQ-14: Is surgical resection recommended in cases with locally recurrent rectal cancer?

- Resection is recommended for local recurrence of rectal cancer when R0 resection is considered possible (Recommendation 2/Evidence level C)

The indication of resection should be decided after considering the surgical stress, risk, and postoperative quality of life.

It is necessary to fully consider the proficiency of the individual surgical team if pelvic exenteration and bony pelvic wall resection are expected.

CQ-15: is postoperative adjuvant chemotherapy recommended for Stage III colorectal cancer?

① Oxaliplatin combination therapy is recommended for Stage III colon cancer (Recommendation 1/Evidence level A)
② Fluoropyrimidine monotherapy is recommended for Stage III colon cancer (Recommendation 2/Evidence level A)

Treatment selection according to recurrence risk 

Comments

In postoperative adjuvant chemotherapy for Stage III colon cancer, oxaliplatin (OX) combination therapy reduces the relative risk of relapse/death by approximately 20% in comparison to 5-FU + *l*-LV, which has been reproducibly confirmed by three RCTs in Europe and the United States [142, 143, 298–300]. Thus, it is recommended as the most effective treatment option.

On the other hand, in an integrated analysis of three randomized controlled trials in Europe and the United States targeting Dukes’ B and Dukes’ C, 5-FU +* l*-LV was associated with significantly better relapse-free survival and overall survival in comparison to surgery alone [301]. Subsequently, in domestic and international randomized controlled trials, the non-inferiority of Cape (X-ACT [302]) and UFT + LV (NSABP C-06 [303], JCOG 0205 [144]) to 5-FU +* l*-LV was shown, followed by the non-inferiority of S-1 to UFT + LV (ACTS-CC [145]). From these facts, it is considered that each of the above-mentioned fluoropyrimidine monotherapies (i.e., 5-FU +* l*-LV, Cape, UFT + LV, S-1) has a survival benefit in comparison to surgery alone. However, its effect has been shown to be inferior to OX combination therapy, as described above.

Upon selecting the actual treatment regimen, the risk of recurrence and the expected effect in each patient should be considered (see above figure). In addition, adequate information, such as adverse events, treatment costs, and hospital visits, should be provided to each patient. It is desirable to select therapy based on comprehensive judgment, including the patient’s general condition and willingness to treat.

CQ-16: Is the recommended duration of postoperative adjuvant chemotherapy 6 months?

① It is recommended that postoperative adjuvant chemotherapy should be performed for 6 months (Recommendation 1/Evidence level A)
② However, if CAPOX therapy is used for low-risk colon cancer, it is recommended that postoperative adjuvant chemotherapy should be performed for 3 months (Recommendation 2/Evidence level A)

CQ-17: Is postoperative adjuvant chemotherapy recommended in patients aged 70 or over?

- Even in patients 70 years old or older, postoperative adjuvant chemotherapy is recommended if their PS is good, if the function of major organs is adequate, and if there are no complications that may be a risk for performing chemotherapy (Recommendation 1/Evidence level A)

CQ-18: Is postoperative adjuvant chemotherapy recommended for Stage II colorectal cancer?

① Postoperative adjuvant chemotherapy is recommended for high-risk Stage II patients (Recommendation 2/Evidence level B)
② However, postoperative adjuvant chemotherapy is not recommended for other Stage II patients (Recommendation 2/Evidence level B)

CQ-19: Is adjuvant chemotherapy recommended subsequent to the resection of a distant metastatic lesion?

① Adjuvant chemotherapy is recommended for patients after the curative resection of liver metastases (Recommendation 2/Evidence level B)
② Adjuvant chemotherapy is recommended for patients after curative resection of distant metastases other than liver metastasis (e.g., lung metastases) (Recommendation 2/Evidence level D)

CQ-20: Is concomitant therapy with molecular targeted drugs recommended as a first-line therapy?

- Usage in combination with either Bevacizumab or anti-EGFR antibody drug is recommended (Recommendation 1/Evidence level A)

Comments

The efficacy and safety of combination therapy with molecular targeted drugs as first-line therapy for unresectable colorectal cancer have been demonstrated for bevacizumab (BEV), cetuximab (CET) and panitumumab (PANI). On the other hand, ramucirumab (RAM), aflibercept beta (AFL) and regorafenib (REG) have not been confirmed in first-line therapy, and their concomitant use is not recommended [193, 194, 214].

Recently, in the pooled analysis of six RCTs (FIRE-3 trial, CALGB/SWOG 80405, PEAK, CRYSTAL, PRIME, 20050181) for *RAS* wild-type unresectable colorectal cancer, a correlation between the tumor location (right side or left side) and the therapeutic effect of molecular targeted drugs (BEV or anti-EGFR antibody) was reported [220]. Based on the results of the analysis, anti-EGFR antibody therapies are recommended for *RAS/BRAF* wild-type colon cancer when the primary lesion is on the left side, while BEV is recommended for cases in which the primary lesion is located on the right side [218]. On the other hand, BEV combination therapy is recommended for *RAS* or *BRAF* mutated colon cancer, regardless of the location of the primary lesion [218]. In *BRAF*-mutated colorectal cancer, FOLFOXIRI + BEV combination therapy has shown high efficacy. Thus, FOLFOXIRI + BEV is recommended as the first choice if it can be applied, considering the age, PS and comorbidities of the patient [175].

Taken together, chemotherapy in combination with BEV or anti-EGFR antibody drugs is recommended as first-line therapy for unresectable colorectal cancer, unless contraindicated. For *RAS/BRAF* wild-type, either BEV or anti-EGR antibody drugs should be selected considering the toxicity profile, backbone chemotherapies, patient preference, and primary tumor location. Since the efficacy of anti-EGFR antibody drugs varies according to the *RAS/BRAF* genotype, it is desirable to perform mutation testing of *RAS* and *BRAF* prior to the selection of the first-line therapy.

CQ-21: Is concomitant therapy with molecular targeted drugs recommended as a second-line therapy?

① Usage in combination with an anti-VEGF antibody drug is recommended (Recommendation 1/Evidence level A)
② Usage in combination with an anti-EGFR antibody drug is recommended (Recommendation 2/Evidence level A)

CQ-22: For third or later line treatments, is the administration of Regorafenib or FTD/TPI recommended?

- If a patient becomes non-responsive or intolerant to fluoropyrimidine, oxaliplatin, irinotecan, either drug is recommended as salvage line therapy.

(Recommendation level 1/Evidence level A)

CQ-23: Are immune checkpoint inhibitors recommended for colorectal cancer?

- Anti-PD-1 antibody therapy is recommended for MSI-H unresectable colorectal cancer patient who have undergone previous treatment (Recommendation level 1/Evidence level B)

CQ-24: Is hepatic arterial infusion therapy recommended in cases of liver metastasis?

- When systemic therapy is available, it is recommended that hepatic arterial infusion therapy not be performed for the treatment of unresectable liver metastasis (Recommendation 1/Evidence level C)

CQ-25: Is neoadjuvant therapy recommended for patients with R0 resectable rectal cancer?

① For rectal cancer with a high risk of local recurrence, preoperative chemoradiotherapy is recommended (Recommendation 2/Evidence level B)
② The efficacy of preoperative chemotherapy (without radiation) has not been established. It is recommended that preoperative chemotherapy not be performed (Recommendation 2/Evidence level C)

CQ-26: Is chemoradiotherapy recommended for unresectable locally advanced and locally recurrent rectal cancer without distant metastasis?

- Chemoradiotherapy directed to resection is recommended for cases in which R0 resection is expected to be possible due to tumor shrinkage (Recommendation 2/Evidence level B)

On the other hand, it is considered reasonable to carry out systemic therapy for the purpose of continuous tumor control when resection cannot be expected. Regarding irradiation of local lesions, it is desirable to consider the symptoms, expected effects, and predicted adverse events.

CQ-27: Is stent treatment recommended for obstructive colorectal cancer?

① Stent treatment for symptomatic relief in patients who are not indicated for systemic therapy is recommended as a treatment option with a reduced physical and psychological burden on patients (Recommendation 2/Evidence level B)
② Stent treatment is not recommended for patients who are indicated for systemic therapy (Recommendation 2/Evidence level B)
③ Obstruction relief by stent treatment as a bridge to surgery (BTS) premised on curative surgical removal avoids emergency surgery and reduces the risk of postoperative complications. However, it is also pointed out that perforation and other adverse effects may worsen the long-term prognosis (No recommendation/Evidence level C)

CQ-28: Is the surveillance of multiple primary cancers (multiple colorectal cancer or other organ cancer) recommended after curative surgery for colorectal cancer?

① Metachronous colorectal cancer occurs more frequently in cases of colorectal cancer resection than in the general population and, as such, regular colonoscopic examination of the large bowel is recommended (Recommendation 1/Evidence level B)
② There is no indication that post-surgical surveillance targeting multiple primary cancers in other organs is effective. As such, it is recommended that no examination be conducted for this purpose. The appropriate course of action is to educate the patient regarding the need for regular cancer examinations and to recommend that they undergo such examinations (Recommendation 2/Evidence level C)
  For hereditary colorectal cancer, it is necessary to carry out surveillance for multiple primary cancers under appropriate counseling (see “JSCCR Guidelines 2016 for the Clinical Practice of Hereditary Colorectal Cancer”) [227].
